# Solid Wastes Provide Breeding Sites, Burrows, and Food for Biological Disease Vectors, and Urban Zoonotic Reservoirs: A Call to Action for Solutions-Based Research

**DOI:** 10.3389/fpubh.2019.00405

**Published:** 2020-01-17

**Authors:** Amy Krystosik, Gathenji Njoroge, Lorriane Odhiambo, Jenna E. Forsyth, Francis Mutuku, A. Desiree LaBeaud

**Affiliations:** ^1^Division of Infectious Disease, Department of Pediatrics, School of Medicine, Stanford University, Stanford, CA, United States; ^2^School of Public Health, University of California, Berkeley, Berkeley, CA, United States; ^3^College of Public Health, Kent State University, Kent, OH, United States; ^4^School of Earth Sciences, Stanford University, Stanford, CA, United States; ^5^Environment and Health Sciences Department, Technical University of Mombasa, Mombasa, Kenya

**Keywords:** planetary health, infectious disease epidemiology, plastic pollution, vector-borne diseases, urban zoonoses, solid waste

## Abstract

**Background:** Infectious disease epidemiology and planetary health literature often cite solid waste and plastic pollution as risk factors for vector-borne diseases and urban zoonoses; however, no rigorous reviews of the risks to human health have been published since 1994. This paper aims to identify research gaps and outline potential solutions to interrupt the vicious cycle of solid wastes; disease vectors and reservoirs; infection and disease; and poverty.

**Methods:** We searched peer-reviewed publications from PubMed, Google Scholar, and Stanford Searchworks, and references from relevant articles using the search terms (“disease” OR “epidemiology”) AND (“plastic pollution,” “garbage,” and “trash,” “rubbish,” “refuse,” OR “solid waste”). Abstracts and reports from meetings were included only when they related directly to previously published work. Only articles published in English, Spanish, or Portuguese through 2018 were included, with a focus on post-1994, after the last comprehensive review was published. Cancer, diabetes, and food chain-specific articles were outside the scope and excluded. After completing the literature review, we further limited the literature to “urban zoonotic and biological vector-borne diseases” or to “zoonotic and biological vector-borne diseases of the urban environment.”

**Results:** Urban biological vector-borne diseases, especially *Aedes-*borne diseases, are associated with solid waste accumulation but vector preferences vary over season and region. Urban zoonosis, especially rodent and canine disease reservoirs, are associated with solid waste in urban settings, especially when garbage accumulates over time, creating burrowing sites and food for reservoirs. Although evidence suggests the link between plastic pollution/solid waste and human disease, measurements are not standardized, confounders are not rigorously controlled, and the quality of evidence varies. Here we propose a framework for solutions-based research in three areas: innovation, education, and policy.

**Conclusions:** Disease epidemics are increasing in scope and scale with urban populations growing, climate change providing newly suitable vector climates, and immunologically naïve populations becoming newly exposed. Sustainable solid waste management is crucial to prevention, specifically in urban environments that favor urban vectors such as *Aedes* species. We propose that next steps should include more robust epidemiological measurements and propose a framework for solutions-based research.

## Introduction

### Rationale

The world is in a solid waste and plastic predicament (1–15)—single use-plastic packaging is increasing in an urbanized (11, 16–20) and globalized economy in which production of food happens farther from the consumer and packaging enables consumption far from the source; yet, plastics lack a circular economy (21–23) that would incentivize responsible management (3, 24–28), resulting in large accumulations of solid waste, specifically plastics which do not biodegrade (29).

The most common approach to eliminating accumulated trash in low- and middle- income countries is open burning. For example, in sub-Saharan Africa, more than 75% of waste is openly burned and worldwide an estimated 600 million tons are openly burned annually (30). Open burning of trash is dangerous for human health (6, 31–33) and the planet, as burning releases toxins into the air that pollute the environment and increase greenhouse gases which contribute to climate change (30).

Policies are slowly catching up to reduce single-use plastic supply, but these policies are only one part of a complete solution (34–37) to single-use plastic production, demand, and disposal (29) and these policies often face poor enforcement, especially in LMICs (29).

At the same time, the risk of zoonosis has increased with urbanization (38) and immunologically naïve populations are newly at risk for vector-borne disease transmission due to changing geographies of suitable vector climates (39–41). Vector-borne diseases such as dengue—transmitted by container breeding *Aedes* spp.—threaten about half a billion people in densely populated areas (42). One very important mosquito vector, *Aedes aegypti*, which spreads dengue, Zika, chikungunya, and yellow fever, prefers to breed in man-made containers (43, 44), such as recyclable plastic containers, tires, and trash. The 2,050 projections of over 6 billion people living in urban areas (45) suggest an impending increase in the risk of infectious disease transmission.

### Objectives

Trash accumulation has been cited as a risk factor for infectious disease (46–50). Recent viewpoints discuss the subject (51–53), but analytical reviews are outdated (54–57). Other reviews exclude key references on trash and disease risk (19, 58–60), while others focus on urbanization or poverty (18, 19, 57). Some reviews take a narrow scope and are pathogen-specific [for example, we identified reviews on trash and dengue virus (61–63), protozoans (64), and leishmania (65)] or vector-specific [arthropods (51)], limited to landfills and incineration (66), microplastic-specific (67), or waste-specific (68). However, the potential risk of direct transmission of infectious diseases by any kind of solid waste depends on a multitude of inter-related factors including, but not limited to, the presence of an infectious agent, its viability in solid waste, and a susceptible host.

A holistic approach is needed to define the link between vector-borne diseases, urban zoonosis, and solid waste. Here, our objective is to identify research gaps through a review of current evidence on solid waste accumulation, in association with urban zoonosis and biological vector-borne disease risk, and to propose solutions that can interrupt the vicious cycle of solid waste accumulation and human health risks due to infectious diseases.

### Research Question

We hypothesize that plastic pollution, including unused plastic bottles, containers, and tires, is a major environmental health risk and promotes vector-borne diseases (VBD) such as dengue, chikungunya, Zika, malaria, and other vectors of disease (triatomine, houseflies) and zoonotic reservoirs (rodents and canines).

## Methods

### Search Strategy

We conducted a hypothesis-driven review from January to March 2019. Literature was identified by searches of PubMed, Google Scholar, and Stanford Searchworks, and references from relevant articles using the search terms (“disease” OR “epidemiology”) AND (“plastic pollution,” “garbage,” and “trash,” “rubbish,” “refuse,” OR “solid waste”). Abstracts and reports from meetings were included only when they related directly to previously published work. Only articles published in English, Spanish, or Portuguese (translated using https://www.deepl.com/translator) through 2018 were included, with a focus on post-1994 (the publication year of the last rigorous review on the topic). Cancer, diabetes, mechanical vectors, and food chain-specific articles were outside the scope of this review and excluded. The search was not constrained by geography. After completing the literature review, we further limited the literature to only biological vector-borne and zoonotic diseases (see general concepts defined in the Supplementary Material).

## Synthesized findings: published literature on vector-borne diseases, urban zoonosis, and solid waste

One hundred and fifty three references were identified in the literature review, 73 of which discussed vector-borne and zoonotic diseases. We discuss vector-borne diseases and urban zoonosis in the context of solid waste and highlight major vectors, reservoirs, and diseases.

We identified 45 references related to vector-borne disease risk and solid waste. We categorized the results according to vector [*Aedes* species, *Phlebotomus* spp. (sand flies), triatominae, *Anopheles* species], pathogen (dengue, chikungunya, and Zika viruses, *Leishmania, Trypanosoma cruzi*, and *Plasmodium*) and type of evidence (case study, observational, intervention, policy, or review). We summarize the evidence in Table 1 and details are available in Supplementary Material 1.

**Table 1:** Vector-borne disease evidence.

**Study type**	**Sample size**	**Year**	**Study site**	**WHO Region (69)**	**Trash type/risk measured**	**References**
**Aedes: DENV**						
**Serosurveys**						
	106 households (501 residents)	2000	El Salvador	Americas	Discarded cans, plastic containers, tire casings	(70)
	273 people	2008	Texas-Mexico border	Americas	Waste tires and buckets	(43)
	600 people	2004	Brownsville, Texas, and Matamoros, Tamaulipas, México	Americas	Water-holding containers, garbage collection	(71)
Focus groups	59 people	2003	San Juan, Puerto Rico	Americas	Insufficient garbage removal	(72)
**Surveillance system studies**						
Case-control study	34 cases and 34 controls	2001	Fortaleza (north-east Brazil)	Americas	No waste collection	(73)
Observational study	219 (139 with and 80 without infection)	2017	Machala, Ecuador	Americas	Daily garbage collection	(74)
**Surveillance system modeling studies**						
	4,165 households	2014	Thailand	S-E Asia	Outdoor solid waste disposal	(75)
	4,248 cases	2018	Guayaquil, Ecuador	Americas	Negative association: municipal garbage collection at the census block level	(76)
Population-based case-control study	538 clinical cases and 727 controls	2011	Campinas, São Paulo, Brazil	Americas	Frequency of garbage collection	(77)
Longitudinal models	165 cases; 492 controls	2018	Fortaleza, Brazil	Americas	Irregular garbage collection, scrapyards and sites associated with tires	(44)
Case-control study	165 cases; 492 controls	2014	Guangzhou, China	Western Pacific	Removing trash and stagnant water from around the residence	(78)
**Entomological surveys**						
Larval	70 clusters; 1,750 houses	2014	Thiruvananthapuram, Kerala, India	S-E Asia	Tires and containers	(79)
Larval	789 breeding habitats	2008–2009	Malaysia	Western Pacific	Plastic containers as breeding habitats	(80)
	205 households	September 2017	Five streets in urban Chidambaram, Cuddalore district, Tamil Nadu state, India	S-E Asia	Discarded plastic containers	(81)
Larval	347 DF/DHF cases in 120 study sites	July 2002–August 2003	Kandy District, Sri Lanka	S-E Asia	Tires, discarded plastic	(82)
**Intervention studies**						
Modeled a hypothetical sanitation program		1999	Montrose urbanization in Caroni County and Port Cumana in the St. Andrews/St. David district, Trinidad	Americas	No effect: tires and small miscellaneous discarded trash	(83)
Waste disposal act		1988–1993	Taiwan	Western Pacific	Discarded containers	(84)
Household level waste management intervention for vector control and community mobilization	200 houses	2012	Gampaha district of Sri Lanka	S-E Asia	Waste management at household level, the promotion of composting biodegradable household waste, raising awareness on the importance of solid waste management in dengue control and improving garbage collection bowls, tins, bottles	(85)
Community-centered dengue-ecosystem management		2012	Yogyakarta city, Indonesia	S-E Asia	Solid waste management and recycling	(86)
		2006 and 2011	India, Sri Lanka, Indonesia, Myanmar, Philippines, Thailand	S-E Asia/Western Pacific	Solid waste management, composting and recycling schemes small discarded containers	(87)
**Aedes: ZIKV/CHIKV**						
**Surveillance system modeling studies**						
		2014–2016	Brazil	Americas	Man-made larval habitats and environmental management—water supply/storage and solid waste management as measured by the *Garbage accumulation index* (number of houses with accumulated and uncollected garbage)	(88)
		2018	Brazil	Americas	Reported garbage destination, type of sanitary installation	(89)
**Aedes: CHIKV**						
Policy brief		June 2012	Reunion Island	Africa	Garbage disposal	(90)
***Aedes albopictus***						
**Entomological surveys**						
Larval	3720 premises and 820 local inhabitants	2010	Sant Cugat, Spain	Europe	Premises with solid waste	(91)
Immatures	four city areas	2007	Fortaleza, Ceará, Brazil	Americas	Tires, opened coconuts and small plastic containers	(92)
Larvae	100 homes	2006–2009	Calicut, Kerala, India	S-E Asia	Coconut shells and plastic waste	(93)
**Intervention studies**						
Area-wide management	six 1000 parcel sites; 3 urban; 3 suburban areas	2013	New Jersey, United States	Americas	Tires and trash (plastic bags, soda cans, etc.)	(94)
***Aedes aegypti***						
**Entomological surveys**						
Larval	750 containers; 1,873 larvae	May-June to September-October 2014	Dire Dawa, East Ethiopia	Africa	Discarded tires and artificial water containers in houses and peridomestic areas	(95)
	18 localities	June 2013 to May 2014	Delhi, India	S-E Asia	Solid waste and plastic containers	(96)
Immature	20 sentinel houses in each of 4 study sites	June 2014 to May 2016	rural and urban sites in western and coastal Kenya	Africa	Buckets, drums, tires, and pots	(97)
Temporal dynamics and spatial patterns	17,815 fixed sites	2016	Tartagal, Salta Province, Argentina	Americas	Municipal garbage dump, tire repair shops, and small garbage accumulation sites	(98)
**Intervention studies**						
Community-based larval source reduction campaign		2003	Lautoka, Viti Levu, Fiji Islands	Western Pacific	Tires and drums	(99)
***Aedes*** **spp**.						
**Entomological surveys**						
Vector survey	175 discardable plastic teacups	2003	Coastal district, Ernakulam, in Kerala State, India	S-E Asia	Plastic teacups discarded at tea carts	(100)
Immatures		2012	Delhi and Haryana, India	S-E Asia	Discarded trash, tires and plastic cups at roadside near tea stalls	(101)
Larval	26 types of wastes	2015	Kolkata, India	S-E Asia	Household wastes: earthen, porcelain, plastic, and coconut shells	(102)
Larval	262 containers	2009	University of Malaya, Kuala Lumpur	Western Pacific	Plastic containers, bottles, and cans	(103)
**Sandflies: leishmaniasis**						
case-control	Two large outbreaks of at least 1,000 newly reported cases	2005	Teresina, Brazil	Americas	Regular trash collection	(104)
KAP	3,968 heads of households	2006	Bihar state, India	S-E Asia	Garbage collection	(105)
Retrospective study	Five time periods; 3,252 cases	1990–2014	Rio Grande do Norte, Brazil	Americas	Lack of garbage collection	(106)
**Triatomine: trypanosoma cruzi**						
**Seroprevalence**						
	26 rural communities; 905 households, 2,156 humans, and 333 dogs	January 2005–December 2008	Parroquia San Miguel, Municipio Urdaneta, Estado Lara, Venezuela	Americas	Household disarray (measured as old and/or damaged artifacts accumulated, materials from construction, inadequate cleaning and free rubbish in the home)	(107)
	15 municipalities; 96 villages; 576 dwellings	2017	Sucre State, Venezuela	Americas	Accumulated garbage as measured by method of garbage disposal	(108)
Entomological surveys: mixed modeling approach	Three villages; 308 houses	2013	Yucatan, Mexico	Americas	Cleaning of trash from the peridomicile	(109)
KAP	Three villages; 570, 702, and 416 houses	2014	Yucatan Peninsula, Mexico	Americas	Trash, cardboard, yard cleaning (collecting trash, cutting down plants and grass, and burning trash)	(110)
Entomological surveys:	1,913 arthropod samples	2019	Urmia, Iran	Eastern Mediterranean	Municipal solid waste landfill	(111)
***Anopheles*** **spp.: Malaria**						
Geospatial analysis	450 water samples	2015	Rawalpindi, Pakistan	Eastern Mediterranean	Low rates of solid waste collection system use	(112)

*KAP, Knowledge, attitude, and practice. One study found no effect (103) and one other found a negative association (93)*.

We identified 16 references related to urban zoonosis and solid waste. We categorized the results according to vector (rodent and canine), pathogen (*Orientia tsutsugamushi, Leptospira, Yersinia pestis, Toxoplasma gondii*, and rabies virus) and type of evidence (case study, observational, intervention, policy, or review). We summarize the evidence in Table 2 and details are available in Supplementary Material 2.

**Table 2:** Urban zoonosis evidence.

**Study type**	**Sample size**	**Year**	**Study site**	**WHO Region (69)**	**Trash type/risk measured**	**References**
**Observational studies**						
Surveillance		1984–2011	Marseille, France	Europe	Garbage collection strikes in which garbage is left on the street	(113)
	3,171 slum residents	April 2003 and May 2004	Slum in Salvador, Brazil	Americas	Residence <20 meters from accumulated refuse	(114)
Surveillance	79 autochthonous human cases	2011–2015	Federal District, Brazil	Americas	Public garbage collection service	(115)
Outbreak	87 leptospirosis cases	1996	Western Region of Rio de Janeiro	Americas	Lower access to solid waste collection –% households served by municipal solid waste collection (accumulation of organic wastes, promoting the proliferation of rodents)	(116)
Outbreak	87 leptospirosis cases	1996	Western Region of Rio de Janeiro	Americas	Waste accumulation	(117)
Cross-sectional KAP	257 residents	May and June 2007	Urban slum community in Salvador, Brazil	Americas	Improving trash collection	(118)
Outbreak & hospital-based surveillance	89 confirmed cases. 22 households with index cases and 52 control households located in the same slum communities	2001	Slum communities in Salvador, Brazil	Americas	Trash collections	(48)
Population based case-control study	66 lab-confirmed cases and 125 age and sex-matched healthy neighborhood controls	October 2000 and March 2001	Salvador, Brazil	Americas	no association: Peri-domiciliary trash accumulation (Visual inspection of accumulated trash & continuous presence of household trash within five meters of a residence—proximity to accumulated trash) and municipal waste collection	(119)
**Rodent: scrub typhus (Orientia tsutsugamushi)**						
Observational	2,002 adults		Vientiane City, Laos	S-E Asia	Poor sanitary conditions (presence of rubbish, animal excrement, etc.)	(120)
**Rodent: bubonic plague**						
Observational: case study		1900	Central Sydney, Australia	Western Pacific	Informal solid waste storage sites, solid waste management	(121)
Observational: outbreak study		1995–1998	Mahajanga, Madagascar	Africa	rubbish	(122)
Water studies	22 water samples		Southern Chile	Americas	Debris found around the household areas: buckets, pails, jars, barrels, and old tires	(123)
Water studies			Peruvian Amazon region of Iquitos	Americas	Clearing away garbage in urban areas	(124)
Observational	888 patients reported clinically	1975	Salvador	Americas	Sewage, rats, water, dogs, mud and garbage,	(125)
	236 households		Southern Chile	Americas	Open containers and debris presence of dogs and rodents	(123)
**Canine: toxoplasmosis**						
Observational: serosurvey of humans and dogs	564 households, which included 597 owners and 729 dogs		Urban areas of a major cities, Londrina, southern Brazil	Americas	Yard cleaning frequency, and having a dirty yard	(126)
**Canine: rabies**						
Observational		2005–2016	Lebanon	Eastern Mediterranean	Local garbage crisis: standing accumulated waste	(127)

*One study found no association (119)*.

### Vector-Borne Diseases and Solid Waste

Vector-borne diseases, especially Aedes-borne diseases, are associated with solid waste accumulation in the urban environment, even small cups, and wrappers, but vector preferences vary over season and region. Other vectors are associated with trash as a burrow, source of food, and breeding site.

#### Aedes Species

*Aedes* species mosquitoes prefer to breed in man-made plastic containers (43, 44) and transmit dengue (DENV), Zika (ZIKV), and chikungunya (CHIKV) viruses. *Aedes albopictus* is reported to preferentially breed in solid waste (91), and tires (92), open coconut shells (92, 93) and small plastic containers (92, 93). *Aedes aegypti* prefers to breed in discarded tires (95, 98) and artificial water containers (95); plastic containers (96), solid waste (96, 98), buckets (97), drums (97), tires (97), pots (97), and garbage dumps (98). Both *Aedes albopictus* and *Aedes aegypti* breed in plastic teacups (100, 101), plastic containers (79–82, 102, 103, 128), tires (79, 82, 101), trash (96, 101), bottles (103), and cans (103). However, these associations change seasonally and regionally. During transmission season, *Aedes* prefers solid waste (96) in Delhi, India. During the rainy season in Brazil, *Aedes* prefers tires (92), open coconut shells and small plastic containers (92). In India, breeding preference ratio was highest for tires and container breeding during pre-monsoon (79). Human DENV transmission was strongly associated with irregular garbage collection during low transmission periods/interepidemic intervals (44).

At the household-level, the evidence shows an increase of dengue risk with the presence of cans, plastic containers, tires (70), a lack of consistent garbage collection (44, 71, 73, 74, 77), and with garbage accumulation (75).

CHIKV and ZIKV have also been associated with garbage accumulation in ecological models (88, 89). However, ecological models can be subject to biases and residual confounding (76). In a case study, Krystosik, Curtis (129) used spatial video and Google Street View in Cali, Colombia to create sub-neighborhood risk surfaces compared with routinely reported clinical cases of dengue, chikungunya, and Zika. Ministry of Health officials and Community Health Workers perceived proximity to unplanned urbanizations without solid waste management as a risk factor for dengue, chikungunya and Zika hotspots. Lack of sanitation can be systematic, for example, 80–90% of housing on Reunion Island was built by squatters resulting in the absence of adequate drainage systems for sewage and rainwater and the lack of properly organized garbage disposal and providing breeding grounds for vector-borne diseases, especially CHIKV (90).

Conversely, removing trash and stagnant water from around the residence is protective (78, 84–87, 94, 99), especially when the government acts with intention and the community is consistently mobilized (85, 86). However, results depend on the local ecology of vector breeding (83, 87).

#### Other Vectors

Other vectors use trash as a burrow, source of food, and breeding site. To prevent tick-borne diseases, The US Centers for Disease Control recommends removing old furniture, mattresses, or trash that may give ticks a place to hide (49); however, no other evidence of an association between trash and tick-borne disease was found. Abbasi et al. (111) identified 33 species of arthropods from a Municipal Solid Waste landfill in Urmia, Iran, including medically important species: *Periplaneta americana Linnaeus* (Blattodea: Blattidae) and *Shelfordella lateralis Walker* (Blattodea: Ectobiidae). Ahmad et al. (112) report that malaria was associated with low rates of solid waste collection system use. However, this association was based on geospatial analysis that did not control for potential confounders. Others report that *Anopheles stephensi* also breeds in manufactured containers (130, 131).

Community members in rural India report that visceral leishmaniasis-transmitting sand flies breed in trash (105). In two studies, the risk of visceral leishmaniasis increased in the absence of regular trash collection (104, 106).

In Yucatan Peninsula, Mexico, residents report triatomines, the vectors of *Trypanosoma cruzi*, burrow in accumulated trash, cardboard, and rocks (110). Strong entomological (109, 110) and clinical (107, 108) evidence supports this local perception. Dumonteil et al. (109) conducted entomological surveillance for one year in 38 randomly selected houses and created crude and adjusted models in which they observed a strong association between the practice of cleaning of trash from the peridomicile and house infestation by non-domiciliated *Triatoma dimidiate* (109). Fortunately, similar to *Aedes* interventions, environmental cleanup is associated with decreased risk of triatomine infestation (110). Clinical evidence also supports these findings. *Trypanosoma cruzi* infection seroprevalence in Venezuela was associated with the increase of accumulated garbage (108) and household disarray (measured as old and/or damaged artifacts accumulated, materials from construction, inadequate cleaning and free rubbish in the home) (107). Bonfante-Cabarcas et al. (107) speculate that accumulated garbage favors breeding of *T. cruzi* reservoirs (rats, mice, and opossum) and provides long-term refuge with immediate food sources for insects to reproduce and colonize the house for a long time, increasing the probability of intra-domiciliary vector transmission of *T. cruzi*.

### Urban Zoonosis Associated With Solid Waste

Urban zoonoses, specifically those transmitted by rodent and canine reservoirs, are associated with solid waste, especially when garbage accumulates over time creating burrowing sites and food for reservoirs.

In a review of neglected tropical diseases and their impact on global health and development (50), Hotez states of zoonoses: “Of relevance to the NTDs, the poorest favelas do not benefit from regular garbage collection or sewage treatment, thereby creating excellent niches for rats and stray dogs.” Rodents and canines directly transmit disease of importance to urban zoonosis (123, 125, 132). Solid waste accumulation is an important factor for urban rodent and canine feeding and sheltering strategies (126) and can be used as a proxy in the absence of reliable data on rodent distribution in the city (117, 126). Presence of rubbish increased risk of scrub typhus (120); Toxoplasma infection in owners and their domiciled dogs was associated with dirty yards (126); and the bubonic plague has historically been associated with solid waste (121, 122).

For example, Kassir et al. (127) conducted an observational study to investigate the risk of rabies and the neighboring Syrian war and the local garbage crisis, finding both were concomitant with a notable increase in the number of dog bites and thus possible rabies exposure. The evidence lies in a time-series of data from the Lebanese Ministry of Public Health (LMOPH) Epidemiological Surveillance Unit public database from 2005 to 2016. A sharp increase in reported animal bites was reported post-2013 (1,004 ± 272 vs. 355 ± 145 bites per year). The authors explain:

“The accumulation of wastes in dumpsites led to the declaration of a severe problem in July 2015, and these open garbage dump sites have been previously shown to contribute to the rise in the number of stray dogs which amplifies the number of possible vectors. Garbage dumps are breeding areas of stray dogs, and if they are no longer around, dogs will migrate to other places. This is reflected by the peak in the stray to domestic dog ratio in October 2015, after heaps of garbage had been covering the Lebanese streets for several months. October, in fact, witnesses the beginning of the rain season in Lebanon, and the rainfall in the presence of open garbage dumps leads to the formation of leachate, a polluting by-product of organic matter. This poses both social and environmental problems such as nuisance, diseases and the spread of stray dogs and other harmful animals. This rise in stray dogs increases the possibility both of new vectors as well as new bites. It is noteworthy that this predominance of stray dog bites was only observed in October 2015, while it was not present in either 2013 or 2014. This further strengthens the correlation between the garbage crisis, a special circumstance of October 2015, and the increase in stray dog bites” (127).

Leptospirosis is associated with dogs (123, 125), accumulated refuse (114), garbage (113, 123) and open containers and debris in the peri-domestic area (123, 125). For example, leptospirosis emergence in Marseille, France is linked to garbage collection strikes that contribute to the expansion of the rat population (113). Among slum residents from Salvador, Brazil, residence <20 m from accumulated refuse was associated with increased odds of previous *Leptospira* infection (114). Residents of another urban slum in Salvador identified improving trash collection as necessary to control leptospirosis in their community and reported current payment for private trash collection service to avoid trash accumulation in their community or a willingness to pay for this service. Residents reported removing trash on a daily basis but identified that trash cans are >50 m from their homes (118). Leptospira interrogans and *L. icterohaemorrhagiae* are pathogens of severe diseases that may cluster in urban areas where trash accumulates (123) but are also found in rural households in peri-domestic open containers (debris found around the household areas including buckets, pails, jars, barrels, and old tires) (123). Evidence shows leptospirosis infection clusters at the household level (48). During a leptospirosis outbreak in Western Rio de Janeiro, Brazil, cases were associated with lower access to solid waste collection, measured as a percentage of households served by municipal solid waste collection (116), and waste accumulation was used as an indicator of probable rat presence (117).

Conversely, in Federal District Brazil, leptospirosis infection was negatively associated with population access to public services: sewage network, treated water network, and public garbage collection services (115); and in Salvador, Brazil, there was no association between leptospirosis infection and peri-domiciliary trash accumulation (119).

### Framework for Solutions-Based Research

Here we propose a framework for solutions-based-research in three areas: innovation, education, and policy.

#### Lessons Learned From Previously Proposed Frameworks

Efforts to promote circular economies in plastics are gaining international attention (29, 133, 134). The United Nations Environment Programme published ‘Single-Use Plastics: A Roadmap for Sustainability, 2018 (29). However, it noted policies and regulations have recently been established and lack monitoring and accountability and suffer from poor implementation. Hawken discusses the short and long term costs and benefits to multiple solutions to Reverse Global Warming (133). However, the solutions require significant investment from business and government to change without a focus on upstream education and innovation. Precious Plastics (134) focuses on the community engagement aspects of reusing plastics but fails to integrate with upstream policy. Examples of successful recycling exist in the metals industry (135–137)—aluminum (135, 136), and steel (137) are recycled and traded as commodities globally.

Perhaps the most common framework is “re-use, reduce, and recycle.” Reusing and recycling receive ample attention given the technology involved, yet trends in the recycling industry are changing: China is no longer accepting foreign trash for recycling (138). Reusing is also challenging as few types of plastics are highly coveted and reusable. The poorer quality plastics are simply trash—unable to be reused or recycled. Therefore, while reusing/recycling/introducing plastic alternatives all have their place, reducing the consumption and sale of single-use plastics is key. Therefore, we are adapting the previously touted framework, emphasizing reduction, and encouraging a circular economy for re-use and recycle.

**Figure 1:**
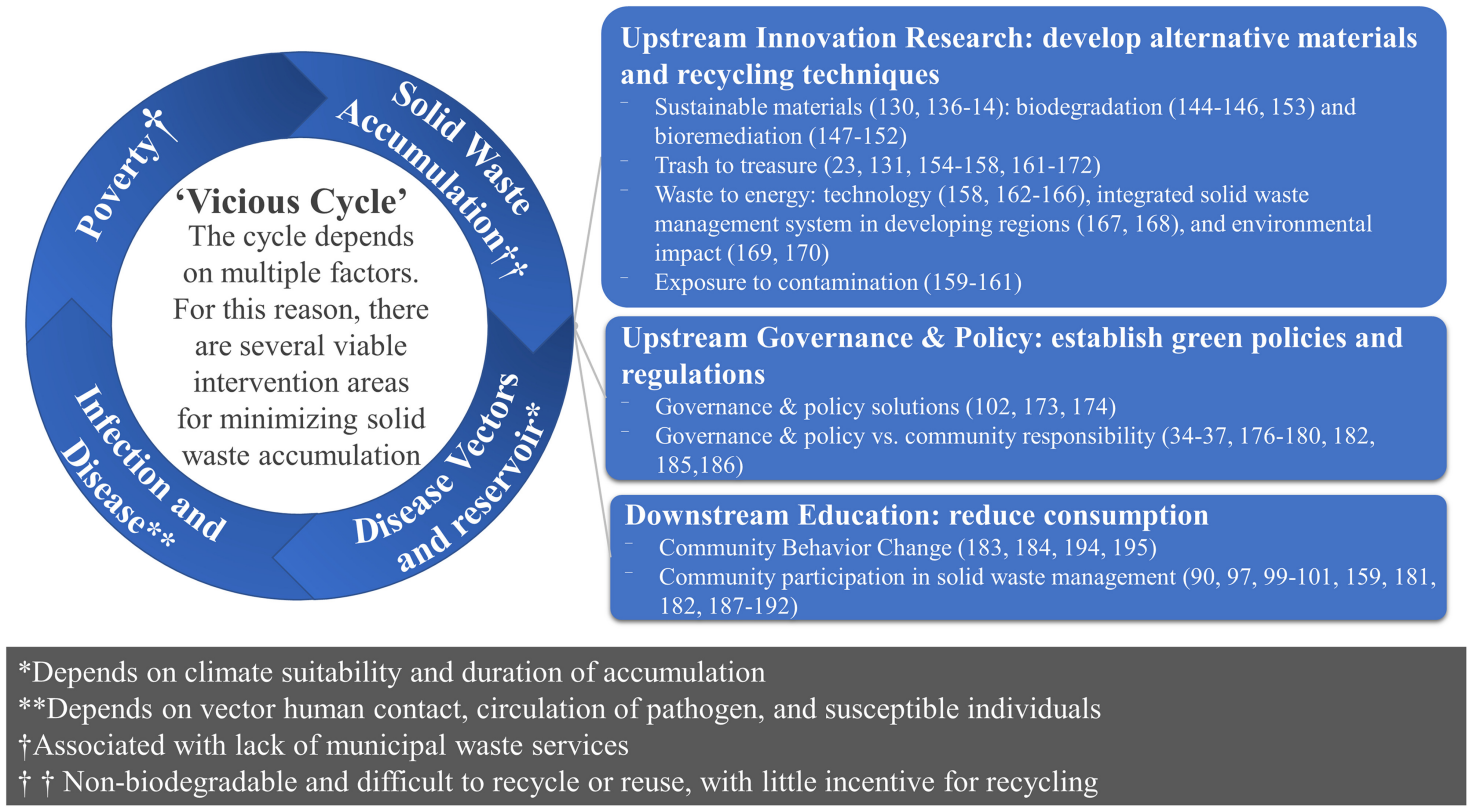
Framework for solutions-based research: upstream innovation research, upstream governance and policy, and downstream education. Recognizing that the current practices and incentive structures that drive these practices are inadequate to address the volume of trash generated globally, different approaches are needed to manage the growing waste problem. Notes for the symbols in the circle. Disease vectors and reservoirs *Depends on climate suitability and duration of accumulation; Infection and Disease **Depends on vector human contact, circulation of pathogen, and susceptible individuals; Poverty is ^†^Associated with lack of municipal waste services; Solid waste accumulation refers to ^††^ Non-biodegradable materials which are not incentivized for recycling.

Building on previous frameworks (29, 133–137), we propose a framework (Figure 1) to reduce vector-borne disease risk and urban zoonoses from exposure to solid waste. Given the importance of intervening at the interface of solid-waste and disease-vectors-and-reservoirs, the framework creates a knowledge-to-action plan using policy and innovative plastic alternatives to decrease the upstream plastic supply, education and art to decrease the downstream global demand for plastic, and innovation to generate profitable uses for currently produced and consumed single-use plastics. The desired result is an action plan to create a circular economy of trash and reduce the supply and demand of single-use plastics and to cultivate empowered, educated, and healthy communities that resist trash accumulation to improve health via reduced vector-borne diseases and improved air quality. The expected impact relates to the critical need to understand how the complex system that generates and discards so much trash might be tweaked, so that less trash is produced or trash is put back into either the economic or ecological cycle. As current options are insufficient, we propose solution-oriented research to either better adapt these options or to create whole new options for plastics disposal, recycling, and reuse and discover possibilities for a future without disposable plastics through policy, education, and innovation. The evidence is summarized in Figure 1 and details are available in Supplementary Material 3.

#### Upstream Innovation Research

Profitable upstream innovation research can decrease supply and improve the processing of solid wastes in an increasingly urbanized and market-based world. Immediate barriers are cost and scalability.

In his 2017 best-seller, Drawdown (133), Hawken discusses the possibility of converting up to 90% of current fossil-fuel based plastic production to bio-based production. However, he warns that the solution must include proper separation and processing to fulfill the goal of sustainable material. Innovation in this field is currently working to drop the price below that of current fossil-fuel-based production. According to a special report commission by the European Polysaccharide Network of Excellence and European Bioplastics, 90% of current plastics could be derived from plants (139). Zhang et al. (140) analyze sustainable materials, defined as a class of materials that are derived from renewable feedstocks and exhibit closed-loop life cycles including aliphatic polyesters and polycarbonates. They also discuss recent advancements that lower the technological barriers for developing more sustainable replacements for petroleum-based plastics including biopolymers (141–143) and agro polymers (144–146).

Two aspects of sustainable materials to consider are biodegradation (147–149) and bioremediation (150–155). Narancic and O'Connor (150–152, 156). We found bioremediation—whereby animals and bacteria can break down plastics into biodegradable products—to be particularly interesting. Narancic and O'Connor (156) review the advances and possibilities in the biotransformation and biodegradation of oil-based plastics, including bio-based and biodegradable polymers, end-of-life management of biodegradables, and a circular economy to reduce plastic waste pollution. New fungi species are biodegrading polyester polyurethane: *Pestalotiopsis* species (150) and *Aspergillus tubingensis* (151). *Ideonella sakaiensis* bacteria break PET (Polyethylene terephthalate) into terephthalic acid and ethylene glycol in 2 weeks (152). Mealworm larvae can digest Styrofoam in <24 h with no cost to survival over 1 month, converting 47.7% of the ingested Styrofoam into CO_2_ and biodegradable residue (153, 154). Wax moth *Galleria mellonella* caterpillars can biodegrade polyethylene bags (155). These methods are especially attractive as they require no behavior change and are sustainable and, in some cases, beneficial to the species performing the biodegradation. Yet, these pilot studies need to be studied at scale and adapted to local context to understand feasibility.

Repurposing trash for profit seems like a viable market-based solution (134, 157–161) but does carry some risk of exposure to contamination (162–164) for entrepreneurs and end-users depending on the type of materials and the processes used and this risk should be taken into consideration early in the process. One popular use case is waste-to-energy analyzes waste-to-energy strategies and concludes that for a net implementation cost of $36 billion, a net operational savings of $19.82 billion and 1.1 gigatons of CO_2_ reduction could be gained. For example, Sweden currently converts 50% of household wastes to energy (161). Yet, Haken warns that this is only a transitional strategy, citing emissions of heavy metals and toxic compounds, even in state-of-the-art facilities. Several reviews discuss waste-to-energy regarding technological options and challenges (165–169), integrated solid waste management in developing countries (170, 171), and the environmental impact (172, 173).

These innovations must come equipped with a knowledge-to-action plan and pilots of these small-scale or theoretical solutions and engagement of external stakeholders such as existing companies, policymakers, and community groups.

#### Upstream Policy

Policymakers are uniquely positioned to make political and normative changes relatively quickly but struggle with enforcement, sustainability subject to elected officials, and community buy-in.

Policymakers are uniquely positioned to prevent and solve public health crises, in collaboration with public health officials and communities (84, 174–176). For example, Chen et al. (84) reported that discarded containers account for 25.4% of *Aedes* vector breeding sites in endemic regions of Taiwan pre-intervention. In 1988, the Waste Disposal Act was amended to make manufacturers, importers, and distributors responsible for the proper recovery, treatment, and recycling of packaging and containers which become an environmental menace. Non-compliance resulted in business suspension. A waste recycling system was established, and a breeding site reduction campaign was promoted for waste management. The authors reported a 98% decrease in dengue incidence reported to the Department of Health from 1988 to 1993. Several countries in Africa continue to implement bans to curb single-use plastic bags which clog drains, sewage systems, or hold rainwater, create breeding grounds for vectors (34).

Experts call for more policy solutions (177–179) and there is evidence that policy agendas can be influenced by popular norms (34, 180). Others argue that informal associations such as waste-picker cooperatives (35, 36, 181, 182) should be strengthened to improve solid waste systems. However, enforcement of such policies may be difficult, especially for nations with challenging processes or non-existent systems (37), and others call for a more community-based approach to increase participation in sustainable waste management (183–185). Businesses that use disposable packaging can also be engaged through social pressure and responsibility to adopt sustainable corporate practices (186, 187) and recoup disposable packaging for recycling.

#### Downstream Education to Decrease Demand

Community-based education and communication have the potential to change norms and create sustainable change but require greater initial investments to tailor and iterate community-based approaches.

Eagle et al. (188) argue that social marketing principles (183, 189, 190) paired with education (75, 85–87, 182, 183, 189–191) and policy (section upstream policy) can intervene to change behavior to positively impact plastic pollution using a transdisciplinary approach to identify barriers to and enablers of sustained behavior change.

Creating awareness about the crisis and health and environmental risks surrounding plastic pollution will not immediately decrease supply, but information may increase social pressure and responsibility to adopt sustainable practices at household (75, 85, 183, 192), community (75, 86, 87, 99, 183, 191, 193–196), and corporate levels (186, 187) that may decrease demand in the future (see details in Supplementary Material 3). For example, Sommerfeld et al. (87) summarize a 5-year research and capacity-building initiative conducted in South Asia and South-East Asia. The initiative developed community-based interventions aimed at reducing dengue vector breeding and viral transmission. Where small discarded containers presented the main problem, groups experimented with solid waste management, composting and recycling schemes. Many intervention tools were locally produced, and all tools were implemented through community partnership strategies. All sites developed socially- and culturally-appropriate health education materials. The study also mobilized and empowered women, students, and community groups and at several sites organized new volunteer groups for environmental health.

Tana et al. (86) built an innovative community-centered dengue-ecosystem management intervention in Yogyakarta city, Indonesia and assessed the process and results. The intervention results included: better community knowledge, attitude, and practices in dengue prevention; increased household and community participation; improved partnership including a variety of stakeholders with prospects for sustainability; vector control efforts refocused on environmental and health issues; increased community ownership of dengue vector management including broader community development activities such as solid waste management and recycling. Tana et al. (86) note, the community-centered approach needs a lot of effort at the beginning but has better prospects for sustainability than the vertical “top-down” approach.

## Discussion

### Summary of Main Findings

Although evidence suggests the link between plastic pollution/solid waste and human disease, measurements are not standardized, confounders are not rigorously controlled, and the quality of evidence varies.

Here we have reviewed the available evidence for solid waste accumulation impact on biological vector-borne diseases. We hypothesized that plastic pollution, including unused plastic bottles, containers, plastic bags, and tires, is a major environmental health risk and promotes vector-borne diseases (VBD) such as dengue, chikungunya, Zika, malaria, and other VBD transmission. We conclude that solid waste accumulation is a risk factor for zoonotic and vector-borne disease transmission. However, measurements are not standardized, (107, 123, 197) and confounders are not rigorously controlled (106, 112, 123, 197, 198).

In the context of vicious cycles of solid waste accumulation, poor health, and poverty, policymakers use estimates of disease transmission, burden, and risk to inform the allocation of limited public health resources; thus, it is imperative epidemiological estimates control for known confounders and employ standardized measurement constructs (Table 3). Additionally, if surveillance data are used, hybrid surveillance (199, 200) should be employed to correct for known surveillance biases. A framework for solutions-based research is also critical to guide research priorities.

**Table 3:** Standardized measurements to define and quantify exposure to solid waste.

**Construct**	**Measurement**	**Unit**	**Covariates**	**Data source**	**References**
Exposure	Distance to accumulated trash	Meters	Frequency of trash collection, size, and type of dump	Local mapping	(98, 114, 117, 119, 129, 182)
	Size of accumulated trash site	Meters	Frequency of trash collection, size, and type of dump	MOH/Local mapping	(117, 129, 182)
	Persistence of accumulated trash	Days	Types of trash	Local mapping	(119)
	Vector breeding in trash	Vector counts	Species, seasonality, infection rates, rainfall, temperature, trash type, trash persistence	Entomological surveys	(79–82, 91–93, 95–98, 100–103, 109, 111)
	Disease Reservoir associated with trash	Reservoir counts	Species, seasonality, infection rates, flooding, food sources, trash type, trash persistence	Animal Surveys	(113, 114, 123, 126, 127)
	Pathogen in trash	Species and concentration	Location, season, container type	Environmental studies	(123, 124)
Access to municipal trash collection	Method of trash disposal	Categorical	Frequency of trash collection, size and type of dump	MOH/Local mapping	(108)
	Population coverage	Percent by region	Distance to trash collection point, cost of service, types of trash accepted	MOH/Local mapping	(116, 117, 119)
	Frequency of collection	Days	Distance to trash collection point, cost of service, types of trash accepted	MOH/Local mapping	(77)
	Distance to trash collection point	Meters	Security of accessing trash collection point	MOH/Local mapping	(129)
	Cost of service	Local monetary unit	Frequency of collection	MOH/Local mapping	(118)
Access to municipal sewage system	Population coverage	Percent by region	Sewage system type (open, closed), distance, cost	MOH/Local mapping	(116)
	Distance to sewage system access	Meters	Rainfall, slope/terrain, manholes, sewage system type (open, closed)	MOH/Local mapping	(114, 129)

*MOH, Ministry of Health*.

Of note, the landscape of single-use plastics innovations and policy is developing rapidly. For example, Christensen et al. described in April 2019 a next-generation plastic to incentivize recycling in closed-loop life cycles (201, 202). This new plastic can be disassembled and reassembled without loss of performance or quality, even in mixed waste streams (201). And the political trend is gaining momentum—in May 2019, 187 countries agreed to add plastics to the Basel Convention, a treaty that regulates the movement of hazardous materials from one country to another (202).

### Limitations

We only included published literature and abstracts in English, Spanish, and Portuguese. We did not have access to primary data and relied on the interpretation of the publishing authors.

Multiple studies included relied on surveillance data which did not correct for selection bias. Multiple studies included did not control for variables possibly associated with both exposure (trash) and outcome (disease), for example, socio-economic status (SES), access to health care, or climate. The data needed to understand the context-specific risk factors are not yet available; particularly, the authors noted a paucity of data from sub-Saharan Africa, where policies and regulations have recently been established (29). Interestingly, although geography was not constrained in the review, most studies identified were from low- and middle-income tropical countries.

After completing our search, we constrained the scope to only urban zoonosis associated with wild mammals and domesticated animals of non-agricultural interest such as dogs and cats. This may exclude some important research related to geographical areas where cows or other domestics animals can serve as crucial reservoirs of important etiological agents.

## Conclusions

Despite gaps in the research base—lack of standardized measures and residual confounding—it is clear solid wastes breed vector-borne diseases and urban zoonoses.

Future populations are at increased risk—disease epidemics are increasing in scope and scale (42) with urban populations growing (38, 45), climate change providing newly suitable vector climates (39–41), and naïve populations becoming newly at risk, sustainable solid waste management is crucial to prevention, specifically in urban environments that favor urban vectors such as *Aedes* species and in poor urban and rural populations which lack access to municipal solid waste services.

We propose a framework for solutions-based research which includes upstream innovation research, upstream policy, and downstream education to decrease demand for single-use plastics.

## Author Contributions

JF, AK, FM, and AL conceived of the initial idea and secured funding. AK drafted the initial manuscript. AK and GN conducted the literature search. LO and AL provided editing on intellectual content. All authors contributed to manuscript revision, read, and approved the submitted version.

### Conflict of Interest

The authors declare that the research was conducted in the absence of any commercial or financial relationships that could be construed as a potential conflict of interest.
