# Supplementary material for: Solid Wastes Provide Breeding Sites, Burrows, and Food for Biological Disease Vectors, and Urban Zoonotic Reservoirs: A Call to Action for Solutions-Based Research
Source: Front Public Health. 2020 Jan 17;7:405. doi: 10.3389/fpubh.2019.00405 (PMC6979070; doi:10.3389/fpubh.2019.00405)
Supplement: Supplementary file 1 [file Data_Sheet_1.pdf]

Supplementary materials

1 Urban Vector borne diseases associated with solid waste

In a review of Neglected Tropical Diseases and their Impact on Global Health and Development [50], Hotez states "The poverty link is based on findings in South Texas and neighboring Mexico regarding the increased risks from the absence of air- conditioning, window screens, and garbage collection and related factors" [50]. In a case study, Krystosik, Curtis [94] used spatial video and Google Street View in Cali, Colombia to create sub-neighborhood risk surfaces compared with routinely reported clinical cases of dengue, chikungunya and Zika. Ministry of Health officials and Community Health Workers perceived proximity to unplanned urbanizations without solid waste management as a risk factor for dengue, chikungunya and Zika hotspots. Risk layers overlapped case density maps but are subject to bias in the routine surveillance data.

1.1.1 Observational

1.1.1.1 Serosurveys

After a dengue-2 outbreak in El Salvador In 2000, Hayes, Garcia-Rivera [85] serosurveyed 106 randomly selected households (501 residents). Odds of infection increased with presence in the home environment of mosquito infested discarded cans (odds ratio [OR] = 4.30, 95% CI = 2.54-7.28 and population attributable fractions [PAF]= 4%), infested discarded plastic containers (OR = 3.98, 95% CI = 1.05-15.05, PAF = 13%), and discarded tire casings (OR = 2.57, 95% CI = 1.09-6.04, PAF = 31%). On the Texas-Mexico border region, M Ramos, Mohammed [43] report results similar to Hayes, Garcia-Rivera [85] where discarded waste tires and buckets were the two largest categories of infested containers. Brunkard, Robles López [86] conducted a cross-sectional serosurvey in Brownsville, Texas, and Matamoros, Tamaulipas, Mexico (n = 600), in 2004 to assess dengue seroprevalence. Authors find "Lack of street drainage appears to limit the ability of mosquito abatement and garbage collection trucks to enter these neighborhoods after a heavy rain. Also, the presence of water-holding containers facilitates vector proliferation in close contact to human hosts."

1.1.1.2 Surveillance system

Perez-Guerra, Zielinski-Gutierrez [201] conducted 11 focus groups (September to October 2003) in San Juan, Puerto Rico with fifty-nine adults (35 women, 24 men) identified through the Puerto Rico dengue surveillance system. Women were concerned more often than men about insufficient garbage removal and water disposal. Suggested strategies to motivate residents' actions included improving access to information on garbage collection and water disposal through telephone hotlines. Heukelbach, de Oliveira [87] conducted a case-control study in a favela in Fortaleza (north-east Brazil) during an outbreak of dengue fever. Cases were defined according to the national guidelines for dengue control as well as based on the detection of IgM-dengue antibodies, and 34 cases and 34 controls were investigated. No waste collection (5.8 (1.1±29.1), P=0.03) was a risk factor for dengue in the unadjusted estimates. Kenneson, Beltran-Ayala [88] noted that unadjusted estimates of daily garbage collection may be protective in households of clinically identified and laboratory confirmed DENV infections in Machala, Ecuador. They called for waste management as part of an integrated community-level prevention program.

1.1.1.3 Surveillance system modeling studies

Campos, Dombrowski [91] conducted an ecological analysis of epidemiological data (2014-2016) on arbovirus co-distribution, environmental and socio-economic factors for each region in Brazil. The Northeast region reported higher number of chikungunya cases, registered the highest number of microcephaly cases, scored lowest on social economic factors, had the highest risk of *Aedes aegypti* house infestation due to the man-made larval habitats, and scored lowest on environmental management—water supply/storage and solid waste management as measured by the *Garbage accumulation index* (number of houses with accumulated and uncollected garbage). The authors conclude the unusual distribution of microcephaly might be due to Zika infection accentuated by poverty and previous or co-infection with other pathogens and reinforces the link between poverty and the risk of disease. However, the analysis is subject to the ecological fallacy.

Suwannapong, Tipayamongkholgul [90] selected one high incidence and one low incidence districts from one dengue endemic province from each region of Thailand. They interviewed heads of households, community members, and local authorities in the highest dengue incidence areas using questionnaires and group interviews and measured household environment. 194 Of 4,561 households reported autochthonous dengue case(s) to local public health staff in the past year. Comparing infected and uninfected households, outdoor solid waste disposal increased household risk of dengue (OR=1.62; 95% CI=1.16-2.29).

Aguiar, Lorenz [92] modeled probable autochthonous ZIK and CHIK cases in 2015 and 2016, along with environmental variables and social indicators. In *Aedes* suitable regions, distribution of ZIKV and CHIKV was predicted by reported garbage destination, type of sanitary installation, and pipe-borne water. Cases were subject to reporting bias as reports of probable autochthonous ZIK and CHIK cases in 2015 and 2016 were obtained from the Notifiable Diseases Information System, which is made publicly available by the Department of the Unified Health System of the Brazilian Ministry of Health

Lippi, Stewart-Ibarra [93] used Local Indicators of Spatial Association, georeferenced dengue cases (n = 4248), and block-level census data variables to identify social-ecological risk factors and hotspots associated with the dengue transmission during outbreak in the city of Guayaquil, Ecuador in 2012. Dengue presence was associated with access to multiple municipal services at the census block level such as garbage collection and piped water. Possible reasons for these counterintuitive result are 1) the census data do not capture the frequency of garbage collection; 2) Municipal services are more widely available in densely populated areas of the city and although access to services should reduce the amount of available habitat for larval mosquitoes, human population density and quality of services may be more important; or 3) Cases included clinically diagnosed and laboratory confirmed cases of dengue fever reported to a surveillance system operated by the MoH as a mandatory notifiable disease yet subject to reporting bias.

Cordeiro, Donalisio [89] conducted a population-based case-control study analyzing 538 clinical cases and 727 controls in one district of the municipality of Campinas, Sao Paulo, Brazil, from 2006-2007 using in-home interviews and inspection of living conditions in and around the homes. Low frequency of garbage collection (OR = 2.6 (95%CI: 1.6-4.5)) was associated with increased incidence of all dengue cases in the multiple binomial regression model controlling for socio-demographic, ecological, case severity, and household infestation variables.

MacCormack-Gelles, Lima Neto [44] used longitudinal models of monthly rates of clinical or laboratory confirmed dengue cases to estimate the differential contribution of contextual factors of epidemic and interepidemic dengue transmission in Fortaleza between 2011 and 2015. Dengue was strongly associated with irregular garbage collection during low transmission periods in models stratified by annual climatological schedules and periods of interepidemic and epidemic transmission, controlling for social, economic, structural, entomological, and environmental factors. They conclude that scrapyards and sites associated with tires (strongly associated with incidence during periods of minimal transmission), require sustained entomological surveillance, particularly during interepidemic intervals and in the urban periphery [44].

Chen, Yang [96] conducted a case-control study during the initial stage of the 2014 DF outbreak in Guangzhou, China. Cases were randomly sampled from the National Notifiable Infectious Disease Reporting Information System (NNIDRIS). Controls were healthy individuals recruited from 17 DF infected communities through cluster sampling. A structured questionnaire on demographics, knowledge, practices, and living environment was administered to participants (165 cases; 492 controls). Logistic regression models identified characteristics of vulnerable populations. Removing trash and stagnant water from around the residence (OR = 0.02, 95% CI = 0.00-0.17) was protective after controlling for demographics, knowledge, practices, and living environment.

1.1.1.4 Vector surveys

Hiriyan, Tewari [76] observed plastic tea cups discarded at tea carts in a coastal district, Ernakulam, in Kerala State, India. They report 36% of 25 water-holding containers were breeding *Aedes albopictus*, suggested that *Aedes albopictus* are competing for the same peri-domestic breeding habitats as *Aedes aegypti*.

Rohani, Aidil Azahary [82] investigated breeding habitats and transovarial dengue virus in *Aedes aegypti* and *Ae. Albopictus* larvae in dengue outbreak areas in Malaysia 2008-2009. Of 789 breeding habitats identified 57.46% were plastic containers. Abramides, Roiz [69] conducted 3720 larval surveys in Sant Cugat, Spain in 2010. *Aedes albopictus* Larval habitats were positively associated with premises with solid waste OR=5.1 (95% CI 3.0-8.9) after controlling for premise type and environmental characteristics.

Getachew, Tekie [73] conducted a cross-sectional immature stage entomological survey in 301 houses from May-June to September-October 2014. of 750 containers (discarded tires and artificial water containers in houses and peridomestic areas) inspected, 405 were positive for mosquito larvae, 84.4% *Aedes aegypti*.

Alencar [70] describe the determinants of immature *Aedes albopictus* infestation in four city areas with high vegetation density and presence of natural breeding sites of Fortaleza, Ceará, Brazil in 2007. During the rainy period, tires, opened coconuts and small plastic containers were most common artificial breeding sites, of which tires held the greatest density.

Kusumawathie and Fernando [83] examined all possible breeding habitats from 12,470 houses and collected larvae of *Ae. aegypti* and *Ae. albopictus* from July 2002 to August 2003 in Kandy District, Sri Lanka. Of 884 positive containers, 12% were tires and 12% discarded plastic containers. They call for effective garbage collection and proper disposal by the local government body for dengue control control in the Kandy District.

Jain, Kushwah [77] piloted an entomological survey of field populations of *Aedes* immature (larvae and pupae) in 2012, over a period of six months from selected sites in Delhi and Haryana, India. Fixed collection spots comprising both domestic and peri-domestic areas were identified, and all collections were made from these spots. Water storage containers in the collection spots included discarded trash, discarded tires and discarded plastic cups at roadside near tea stalls.

Vijayakumar, Sudheesh Kumar [81] determine the larval diversity, density, and breeding site preferences of *Aedes* mosquitoes, during pre-monsoon and monsoon periods in urban and rural areas of Thiruvananthapuram district in the state of Kerala, India. They conducted WHO standard larval surveys in 70 dengue clusters in domestic and peri-domestic areas of 1750 houses. Breeding preference ratio was highest for tires and container breeding was highest pre-monsoon.

Banerjee, Aditya [79] assessed household wastes as *Aedes* spp. larval habitats in Kolkata, India including earthen, porcelain, plastic, and coconut shells. *Aedes* immature density varied by waste containers type and months. They report that most frequently disposed containers sustained *Aedes* spp. population and call for a strict legislation towards disposal and enhanced management of household wastes. In 2015, they surveyed *Aedes spp* larval productivity in urban and rural Kolkata, India and observed plastic containers to be more productive in urban areas compared to rural after controlling for material and season [80].

Rao and George [71] conducted entomological surveys in 100 homes from 2006-2009 in Calicut, Kerala, India, and report. *Ae. albopictus* larvae were most often in coconut shells and plastic waste.

Vikram, Nagpal [74] conducted entomological surveys in Delhi from June 2013 to May 2014. Solid waste was the preferred breeding site during transmission season. overhead tanks (OHTs) and curing tanks were preferred breeding containers during non-transmission season. Plastic containers (29%) in low income group (LIG); solid waste (27%) and plastic containers (26%) in Medium Income Group (MIG); and solid waste (27%) and curing tanks (21%) in High Income Group (HIG) were the most preferred breeding containers for the breeding of Aedes aegypti.

Ngugi, Mutuku [75] characterized breeding habitats and establish container productivity profiles of Ae. aegypti in rural and urban sites in western and coastal Kenya by surveying for immature mosquito infestation once a month for a period of 24 months (June 2014 to May 2016) in twenty sentinel houses in each study site. All water-holding containers in and around the households were inspected for Ae. aegypti larvae and pupae. Of 17,537 Ae. aegypti immatures sampled from 10 container types, the most important habitat types were buckets, drums, tires, and pots, which produced over 75% of all the pupae.

Chena, Leeb [78] surveyed container breeding sites of Aedes spp. and Culex spp. larvae in the campus of the University of Malaya, Kuala Lumpur. Of 262 containers identified only 65 containers contained larvae. Among all types of containers, 33% of plastic containers, 15% of bottles, and 13% of cans were positive with mosquito larvae.

Boornema and Senthil Murugan [84] identified preferred *Aedes* spp. breeding habitats and asses dengue knowledge and awareness in a cross-sectional study in September 2017 on five streets in urban Chidambaram, Cuddalore district, Tamil Nadu state, India. Amongst 205 households surveyed, discarded plastic containers were the major outdoor breeding habitat.

1.1.1.5 Vector survey modeling studies

Espinosa, Weinberg [72] included municipal garbage dump, tire repair shops, and small garbage accumulation sites as critical breeding sites in their model of Temporal Dynamics and Spatial Patterns of Aedes aegypti Breeding Sites, in the Context of a Dengue Control Program in Tartagal, Salta Province, Argentina. These critical breeding sites were classified based on elevated number of containers in comparison to those registered in the housing units.

1.1.2 Intervention

Raju [97] evaluated a Community-based *Aedes aegypti* larval source reduction campaign in a peri-urban district of Lautoka, Viti Levu, Fiji Islands by analyzing the vector surveillance program of the Ministry of Health. 51% of tires and 21% of drums contained *Aedes aegypti* pre-intervention and Breteau indices dropped from 29 in January to below five in nine months post-intervention. Fonseca, Unlu [98] evaluated an area-wide management of *Aedes albopictus* on six 1000-parcel sites, three in urban and three in suburban areas of New Jersey, United States. A total of 19,121 containers were either treated or removed from the full intervention site, including 533 tires and 5792 trash items (plastic bags, soda cans, etc.). They report even small and shallow containers (e.g. snack bags) produced adults.

1.1.2.1 Modeling

Focks, Brenner [103] modeled a hypothetical sanitation program designed to eliminate abandoned tires and small miscellaneous discarded trash. The authors report a sanitation program would be expected to have variable results depending on local ecology of vector breeding. For example, in the Montrose urbanization in Caroni County, tires and trash containers not important vector breeding sites. In Port Cumana in the St. Andrews/St. David district, tires and trash containers accounted for >60% of all *A. aegypti* production. Expected reductions in pupae per person and probability of transmission reflect the local vector ecology. They conclude that a sanitation program, even if completely effective, would not result in prophylactic levels of dengue vectors anywhere in Trinidad, because all areas continue to be above threshold levels. The authors support tailoring control efforts on a site-by-site basis due to site differences in the types, frequencies, and productivities of water-holding domestic and trash containers.

1.1.2.2 Dengue

Chen, Hwang [102] reported that discarded containers account for 25.4% of *Aedes* vector breeding in endemic regions of Taiwan pre-intervention. In 1988, the Waste Disposal Act was amended to make manufacturers, importers, and distributors responsible for the proper recovery, treatment, and recycling of packaging and containers which become an environmental menace. Non-compliance resulted in business suspension. A waste recycling system was established, and a breeding site reduction campaign was promoted for waste management. The authors reported a 98% decreased in dengue incidence reported to the Department of Health from 1988 to 1993.

Abeyewickreme, Wickremasinghe [99] assessed a community mobilization and household level waste management intervention for *Aedes* vector control in Gampaha district of Sri Lanka. Entomological and sociological surveys at 3 months, at 9 months and at 15 months post-intervention revealed that waste management with the elimination of the most productive water container types (bowls, tins, bottles) led to a

significant reduction of *Aedes* pupal indices as a proxy for adult *Aedes* vector densities. The mean *Aedes* Breteau index dropped from 11.75 and 9.75 at baseline to 3.13 and 6.25 in survey round 4 in the intervention and the control clusters, respectively. The study design consisted of eight clusters with roughly 200 houses each selected randomly from high and low dengue endemic areas; 4 intervention arm (2 in the high and 2 in the low endemicity areas) and 4 control arm (2 in the high and 2 in the low endemicity areas)). Entomological and sociological surveys at baseline showed no significant differences in pupal and larval indices of *Aedes* mosquitoes. Intervention clusters received building partnerships of local stakeholders, waste management at household level, the promotion of composting biodegradable household waste, raising awareness on the importance of solid waste management in dengue control and improving garbage collection with the assistance of local government authorities.

Tana, Umniyati [100] evaluated an innovative community-centered dengue-ecosystem management intervention in Yogyakarta city, Indonesia. Entomological surveys and household surveys were conducted at baseline. Six months post intervention, compared to two control neighborhoods, two intervention neighborhoods improved community knowledge, attitude, and practices; increased household and community participation; improved sustainable partnerships; refocused vector control efforts on environmental and health issues; increased community ownership of dengue vector management including solid waste management and recycling.

Sommerfeld and Kroeger [101] describe research and capacity building conducted between 2006 and 2011 in urban and peri-urban areas of six countries of South Asia (India, Sri Lanka) and South-East Asia (Indonesia, Myanmar, Philippines, Thailand). In Sri Lanka and Thailand where small discarded containers presented the main problem, groups experimented with solid waste management, composting and recycling schemes. The programmes reduced vector densities in some sites yet varied due to 'contamination' of control groups.

1.1.3 Policy brief

80-90% of housing on Reunion Island was built by squatters resulting in absence of adequate drainage systems for sewage and rain water and the lack of properly organized garbage disposal and providing breeding ground for vector-borne diseases, especially chikungunya. Aoustin [95] describe a new law enacted in June 2012 to provide a legal basis to public officials to act against urban sprawl and unsanitary housing conditions in French overseas departments with support of French or European courts.

1.2 Sandflies

1.2.1 Observational study

Werneck, Rodrigues [109] conducted a case-control study of 200 visceral leishmaniasis positive households in Teresina, Brazil, where, since 1980, there have been two large outbreaks of VL. They report the risk of acquiring visceral leishmaniasis was higher amongst those with inadequate sewage system (OR = 1.47–11.80) and those without regular trash collection (OR = 1.66–21.60) after controlling for age, crowding, and the background incidence of VL in the area where the subjects lived. Singh, Reddy [108] investigated Knowledge, attitude, and practices related to leishmaniasis in a rural area of Bihar state, India. The survey included 3,968 heads of households in a rural area, of which 63.6% reported the vector breeding site was garbage collection. Lima, Lima [110] conducted a retrospective study of human VL in the state of Rio Grande do Norte, Brazil, for the period of 1990-2014 and report that VL was more frequently found in areas that lacked urban infrastructure, detected by lack of garbage collection and sewers. However, how these estimates were adjusted for potential confounders is unclear in the methods section.

1.3 Triatomine

We found four observational studies regarding Chagas' seroprevalence and knowledge attitudes and practices and triatomine entomology.

1.3.1 Knowledge attitudes and practices

Rosecrans, Cruz-Martin [111] investigated knowledge, attitudes and practices surrounding Chagas' disease, triatomines and vector control in three rural communities in the Yucatan Peninsula, Mexico using qualitative

and quantitative methods. Authors report community members are knowledgeable about triatomines habits. Community members were quoted saying:

> Male 1: ‘‘Under rocks in the bushes. Also in houses where there is lots of trash… under rocks. Yes, where there is lots of trash, cardboard.”
>
> Female 1: ‘‘Yes, for example if we collect rocks and make a pile and sometimes they make their little nest there and live there.’’

Authors report yard cleaning (collecting trash, cutting down plants and grass, and burning trash) is perceived as desirable and effective. Community members were quoted saying:

> Male 4. ‘‘Clean the vegetation, like for example the weeds, the trash, the rocks, collect them together so that bugs don’t have a place to hide.’’
>
> Male 5. ‘‘Like most bugs, because when they see it’s clean they don’t like it, they like it where there is trash.’’

1.3.2 seroprevalence

Bonfante-Cabarcas, Rodríguez-Bonfante [114] and Garcia-Jordan, Berrizbeitia [113] describe seroprevalence. Garcia-Jordan, Berrizbeitia [113] surveyed 96 villages and 576 dwellings in 15 municipalities of Sucre State, Venezuela. Risk factors associated with T. cruzi infection seroprevalence included accumulated garbage as measured by method of garbage disposal (adjusted OR=3,2 1,29-7,74 p=0,008). Authors note that garbage increases the risk of contracting the T. Cruzi infection, because it favors the breeding of rats, mice and rabipelados which are reservoirs of T. cruzi. However, the risk factor associated with seroprevalence was reported burying garbage. The authors reason that it is possible that respondents provided false or misleading information about final garbage disposal. The authors assume that the garbage is not completely buried, but is exposed or exposed to the elements, which provides a refuge for insects to reproduce and colonize the house for a long time, increasing the probability of vector transmission. Bonfante-Cabarcas, Rodríguez-Bonfante [114] serosurveyed 26 rural communities including 905 households, 2,156 humans, and 333 dogs in Parroquia San Miguel, Municipio Urdaneta, Estado Lara, Venezuela, January 2005-December 2008. Previous Trypanosoma cruzi infection was associated with household disarray (measured as old and/or damaged artifacts accumulated, materials from construction, inadequate cleaning and free rubbish in the home) (OR=4,08, 2,17-7,66). The authors explain that this long-term refuge with immediate food sources has allowed the vectors to survive and colonize homes and increase the probability of intradomiciliary vector transmission of Chagas.

1.3.3 Entomology

Dumonteil, Nouvellet [112] conducted entomological surveillance in 308 randomly selected houses for one year in three rural villages in Yucatan, Mexico. The authors used a mixed modeling approach based on initial null-hypothesis testing followed by multimodel inference and averaging on data. of 127 eco-bio-social variables describing in detail housing structure and characteristics (34 variables), peridomicile structure and characteristics (56 variables), socio-demographic characteristics and cultural practices (38 variables), the practice of cleaning of trash from the peridomicile (OR = 2.68 [1.19–6.04]) was one of the strongest five determinants for house infestation by non-domiciliated Triatoma dimidiate in crude and adjusted models [112].

1.4 **House flies**

Alameda County Vector Control [203] warns residents, “Yard or construction wastes, such as decomposing grasses will provide breeding sources for lesser houseflies that hover under covered porches throughout a neighborhood, creating a significant nuisance” And Chaiwong, Srivoramas [204] states “The Oriental latrine fly, Chrysomya megacephala (Fabricius) (Diptera: Calliphoridae) and the house fly, Musca domestica L., (Diptera: Muscidae) are synanthropic flies which are adapted to live in close association with human habitations, thereby making them likely mechanical vectors of several pathogens to humans.”

1.4.1 Observational

Chaiwong, Srivoramas [204] collected 7,750 flies (6 401 C. megacephala and 1 349 M.domestica) by entomological surveillance (monthly from September 2010 to October 2011) from fresh-food markets, garbage piles, restaurants, school cafeterias and paddy fields, in the Muang Ubon Ratchathani and Warinchamrap districts of Ubon Ratchathani province of Northeast Thailand. From 994 flies, authors isolated 15 bacterial genera from the external surfaces: coagulase-negative staphylococci, Streptococcus group D non-enterococci, Salmonella sp., Shigella sp., Escherichia coli O157:H7, Salmonella typhi, Bacillus sp., and Enterococcus sp., Staphylococcus aureus and Pseudomonas aeruginosa. compared to M. domestica, C. megacephala were more commonly bacteria positive (99.28 versus 90.43%) and carried ~11-12 times greater bacterial load. Nazni, Seleena [205] baited flies at breeding sites including dumping ground in Peninsular Malaysia and isolated Bacillus sp., Coccobacillus sp., Staphylococcus sp., Microccus sp., Streptococcus sp., Acinetobacter sp., Enterobacter sp., Proteus sp., Escherichia sp., Klebsiella sp. and yeast cells from feaces, vomitus, external surfaces and internal organs of house fly, Musca domestica. From July to September 2015 in Hamadan, Iran, [206] conducted an entomological cross-section survey, collecting 75 house flies from accumulated of garbage at each of the following: two educational hospitals, a fruit and vegetables center, and a livestock slaughter. Authors isolated 394 bacterial strains from 275 flies, the most common being Bacillus spp (31.1%), Staphylococcusspp (22.9%), and Escherichia coli (11.6%). Bacteria extracted from house flies from the hospital environments were more resistant to antibiotics compared to non-hospital environments. The maximum bacterial isolation was found in houseflies from hospital No.1 environment from around the accumulation of garbage.

In December 2004, a tsunami hit coastal villages on southern parts of Coramandel Coast of India, resulting in seawater inundation and relief camps. Srinivasan, Jambulingam [207] measured muscoid fly abundance and distribution monthly (February 2005 to January 2006) in devastated human settlements, temporary shelters, garbage dumping yards and open defecation yards in seawater intrusion and indoors and outdoors of seawater non-intrusion areas. Authors report a sudden increase in the number of flies associated with seawater incursion, crowding of tsunami victims at relief camps, accumulation of solid waste at centralized relief kitchen and temporary shelters. However, they report no vector-borne disease outbreaks.

1.5 Other

To prevent tick borne diseases, The US Centers for Disease Control recommends removing old furniture, mattresses, or trash from the yard that may give ticks a place to hide [49]. However, no other evidence was found.

Abbasi, Rafinejad [104] identified 33 species of arthropods from a Municipal Solid Waste landfill in Urmia, Iran, including medically important species: Periplaneta americana Linnaeus (Blattodea: Blattidae) and Shelfordella lateralis Walker (Blattodea: Ectobiidae).

Ahmad, Aziz [105] report that malaria was associated with low rates of solid waste collection system use. However, this association was based on geospatial analysis that did not control for potential confounders. Others report that *Anopheles Stephensi* also breed in man-made containers [106,107].

2 Urban zoonosis associated with solid waste

Rodents directly transmit disease of importance to urban zoonosis[115]. Solid waste accumulation as an important factor for urban rodent feeding and sheltering strategies and can be used as a proxy in the absence of reliable data on rodent distribution in the city[119].

2.1 Scrub typhus observational

Vallee, Thaojaikong [120] report 20.3% of randomly selected adults in urban and peri-urban Vientiane City (n = 2,002, ≥35 years) were IgG ELISA seropositive against scrub typhus (Orientia tsutsugamushi). Living on plots of land with **poor sanitary conditions** (presence of rubbish, animal excrement, etc.) increased odds past infection of scrub typhus (OR = 1.7 [1.1–2.7], p= 0.02) controlling for length of residence in Vientiane, education, incomes, households size, and occupation were at higher risk of scrub typhus.

2.2 Leptospirosis: Observational

In a review of Neglected Tropical Diseases and their Impact on Global Health and Development [50], Hotez states "Of relevance to the NTDs, the poorest favelas do not benefit from regular garbage collection or sewage treatment, thereby creating excellent niches for rats and stray dogs."

Socolovschi, Angelakis [125] surveyed all human cases of leptospirosis in Marseille over the last 30 years using clinical, epidemiological, serological, and molecular tools. The conclude leptospirosis emergence is a public health problem in Marseille associated with a combination of heavy rainfall and garbage collection strikes (82 days as reported by local press) in which garbage is left on the street and thereby contributes to the expansion of the rat population on the surface (as trapped in alleys near to where the patients may have acquired leptospirosis). However, it is not clear how the longitudinal nature of the data was accounted for in the analysis or if confounders and spatial relationships were considered.

Reis, Ribeiro [124] estimated 15.4% (95% confidence interval [CI], 14.0-16.8) prevalence of agglutinating antibodies amongst 3,171 slum residents from Salvador, Brazil. In a multivariate model controlling for significant demographic, socioeconomic, household and workplace-related factors, residence less than 20 meters from accumulated refuse was associated with 1.43 times (95% CI 1.04–1.88) increased odds of previous Leptospira infection.

Santos, Landi [128] investigated 79 autochthonous human cases of leptospirosis reported to the public health network (2011-2015). The probable infection location was associated with urban areas during the rainy season, and population access to public services: sewage network, treated water network, and public garbage collection service. However, they did not control for potential confounders such as population density, recall bias of probable infection locations, and reporting bias according to who has access to clinical diagnosis in the public health network.

Barcellos and Sabroza [127] investigated a leptospirosis outbreak in the Western Region of Rio de Janeiro in 1996. census tracts with Leptospirosis cases were associated with lower access to solid waste collection as measured as a percentage of households served by municipal solid waste collection. The absence of this service allows the accumulation of organic wastes, promoting the proliferation of rodents. However, it is not clear how the spatio-temporal aspects of the data were considered in analysis nor the potential confounders. Studying the same outbreak, Barcellos and Sabroza [119] use waste accumulation as an indicator of probable rat presence by region. The authors report that incidence rates were higher for census tracts inside the flood risk area and in the vicinities of waste accumulation sites. However, the authors do not control for potential confounders.

Navegantes de Araujo, Finkmoore [126] conducted a cross-sectional study of 257 residents of an urban slum community in Salvador, Brazil. 17% of respondents identified improving trash collection as necessary to control transmissible disease in their community. 67% reporting willingness to pay for a private trash collection service to avoid trash accumulation in their community,17 of which currently pay someone outside their household to transport their household garbage to the trash can. 56% reported removing trash from their household daily; 68% identified a trash can they used on a regular basis, and 58% said that the nearest trash can was > 50 m far from their homes.

Maciel, de Carvalho [48] identified household clustering of Leptospira infection in slum communities in Salvador, Brazil where recurrent epidemics occur. Using hospital-based surveillance, authors identified 89 confirmed cases of leptospirosis during an outbreak and collected serum samples from members of 22 households with index cases and 52 control households located in the same slum communities. Residence in a household with an index case of leptospirosis was associated with increased risk (OR 5.29, 95% CI 2.13-13.12) of having had a Leptospira infection for all age groups. The authors hypothesize household level clustering is related to proximity to environmental sources of contamination, such as open sewers, flood areas and trash collections; however, they did not measure household level risk factors.

Sarkar, Nascimento [129] conducted a population-based case-control study to identify risk factors for acquisition of leptospirosis in neighborhoods of Salvador, Brazil with high endemicity during the rainy season-associated urban epidemic. Authors interviewed Sixty-six laboratory-confirmed cases and 125 age and sex-matched healthy neighborhood controls. In adjusted analysis, peri-domiciliary sighting of rats (OR = 4.49, 95% CI = 1.57-12.83) and sighting groups of five or more rats (OR = 3.90, 95% CI = 1.35-11.27) were independent risk factors for acquiring disease. Peri-domiciliary trash accumulation (Visual inspection of accumulated trash & continuous presence of household trash within five meters of a residence was defined as "proximity to accumulated trash.") and municipal waste collection were not found to be significant risk factors Using matched univariate analysis, not controlling for potential confounders.

Ganoza, Matthias [208] and Munoz-Zanzi, Mason [117] conducted environmental water studies to identify leptospirosis. Munoz-Zanzi, Mason [117] compared levels of *Leptospira* in urban and rural environmental surface waters in sites in the Peruvian Amazon region of Iquitos using QRT-PCR and identified sequenced RNA to determine *Leptospira* species. Leptospires were present in samples from gutters and puddles in the urban slum's market area at 20 times the concentration of rural sites and *Leptospira interrogans*, icterohaemorrhagiae, most commonly associated with severe disease, was found more often in the urban water samples than in the rural ones. These data suggest that environmental interventions such as reducing sources of standing water and clearing away garbage in urban areas might reduce the number of cases of severe leptospirosis. However, the study did not measure garbage near samples collected nor control for distance to said garbage or potential confounders. Munoz-Zanzi, Mason [117] detected pathogenic Leptospira by PCR in 10/22 water samples from peri-domestic open containers (debris found around the household areas and included, but were not limited to, buckets, pails, jars, barrels, and old tires) from rural households in southern Chile.

2.3 Plague

Two case studies of plague and solid waste offer a historical prospective. Milke [121] describe the solid waste crisis leading up to the bubonic plague outbreak in central Sydney in 1900, stating:

> "Basements of properties became informal solid waste storage sites, sewer connections were either not provided or poorly maintained, and small businesses like butchers ran unsanitary operations with little regard for solid waste management. Rapid urban growth coupled with poor sanitation and the location near docks led to the development of a large rat population in central Sydney and the ability of rats to have close contact with workers and residents. Poor government allowed the situation to develop and– in spite of calls for improvement—worsen."

Similarly, Boisier, Rahalison [122] describe annual outbreaks of bubonic plague (1995 to 1998) in the coastal city of Mahajanga, Madagascar clinically suspected and laboratory confirmed cases concentrated in crowded and unsanitary districts which result in close contact among humans, rats, and shrews, stating: "the epicenter for the two waves of the outbreak, is densely populated with very poor people. This area also includes the two largest markets, which generate the town's largest amount of rubbish."

2.4 Canine: Observational

Canines have also been cited as sentential proxies of diseases transmission. In a review of Neglected Tropical Diseases and their Impact on Global Health and Development [50], Hotez states:

> "In these regions, poor rural housing conditions such as cracked walls and earthen floors allow the sandfly vectors that transmit both Visceral leishmaniasis and Cutaneous leishmaniasis to flourish, while in some urban areas with poor sanitation, the uncollected garbage provides additional sandfly niches, because it promotes habitats for stray dogs, which can sometimes serve as animal reservoirs of the infection."

2.4.1 Leptospirosis

Caldas and Sampaio [116] conducted an observational study of 888 patients reported clinically to Salvador's Infectious Disease Hospital in 1975. 133 patients (36%) were serologically confirmed cases of leptospirosis. In

further epidemiological analysis, the most likely source of infection were sewage, rats, water, dogs, mud and garbage, in that order. Munoz-Zanzi, Mason [117] investigated Leptospira contamination in 236 households and surrounding environmental water in rural communities in southern Chile and reported that the second most commonly collected sample type was from open containers and debris in the peri-domestic area with evidence of contamination, at various levels, in all sample types including containers and debris. Presence of dogs (OR = 15.9, p = 0.022) and rodents (OR = 3.22), especially in low income households (OR = 4.35, p = 0.003), was also positively associated with positive puddles but no direct comparison with trash and dogs or rodents was made.

2.4.2 Toxoplasmosis

Benitez, Martins [118] assessed the seroprevalence (IgG anti-T. gondii antibodies) and associated variables of Toxoplasma infection in owners and their domiciled dogs in in urban areas of a major cities, Londrina, southern Brazil. They conducted representative random sampling among 564 households, which included 597 owners and 729 dogs. Seropositivity of household people and dogs was associated with public sewer service, yard cleaning frequency, and having a dirty yard. Dog seropositivity was associated with having more dogs and a dirty backyard and was also spatially clustered (RR 3.22; p<0.001) in an unplanned urbanization with inadequate infrastructure and semi- to non-domiciled dog populations. They conclude "dogs may be a reliable sentinel for environmental infection. Moreover, such a holistic approach may provide crucial information for more focused prevention and monitoring programs, particularly in households with multiple pets and trash-filled backyards" [118].

2.4.3 Rabies

Kassir, El Zarif [123] conducted an observational study to investigate the risk of rabies and the neighboring Syrian war and the local garbage crisis, finding both were concomitant with a notable increase in the number of dog bites and thus possible rabies exposure. The evidence lies in a timeseries of data from the Lebanese Ministry of Public Health (LMOPH) Epidemiological Surveillance Unit public database from 2005-2016. A sharp increase in reported animal bites was reported post-2013 (1004 ± 272 versus 355 ± 145 bites per year). The authors explain:

> "The accumulation of wastes in dumpsites led to the declaration of a severe problem in July 2015, and these open garbage dump sites have been previously shown to contribute to the rise in the number of stray dogs which amplifies the number of possible vectors. Garbage dumps are breeding areas of stray dogs, and if they are no longer around, dogs will migrate to other places. This is reflected by the peak in the stray to domestic dog ratio in October 2015, after heaps of garbage had been covering the Lebanese streets for several months. October, in fact, witnesses the beginning of the rain season in Lebanon, and the rainfall in the presence of open garbage dumps leads to the formation of leachate, a polluting by-product of organic matter. This poses both social and environmental problems such as nuisance, diseases and the spread of stray dogs and other harmful animals. This rise in stray dogs increases the possibility both of new vectors as well as new bites. It is noteworthy that this predominance of stray dog bites was only observed in October 2015, while it was not present in either 2013 or 2014. This further strengthens the correlation between the garbage crisis, a special circumstance of October 2015, and the increase in stray dog bites" [123].

3 Framework for solutions-based research

3.1 Upstream Innovation Research

3.1.1 Sustainable and biodegradable plastics

In his 2017 best-seller Drawdown[130], Hawken discusses the possibility of replacing up to 90% of current fossil fuel based plastic production to bio-based.[a] However, he warns that the solution must include proper separation and processing to fulfill the goal of sustainable material. Innovation in this field is currently working to drop the price below that of current fossil-fuel based production. According to a special report commission by European Polysaccharide Network of Excellence and European Bioplastics, 90 percent of current plastics could be derived from plants [136]. Zhang, Fevre [137] analyze sustainable materials, defined as a class of materials that are derived from renewable feedstocks and exhibit closed-loop life cycles including aliphatic polyesters and polycarbonates. They also discuss recent advancements that lower the technological barriers for developing more sustainable replacements for petroleum-based plastics.

3.1.1.1 Biopolymers

Pathak, Sneha [138] discuss the history of bioplastics, both biodegradable and non, and the challenges which include higher costs compared to conventional plastics, misconceptions about the environmental costs which still include production of greenhouse gases, and the cost to agriculture for raw materials. Erich, Hannes [139] review the potential for bio-based and biodegradable biopolymer PHB (polyhydroxy butyrate) to replace fossil commodity plastics PP (polypropylene). They conclude that production is increasing (currently less than 0.1% that of PP). Pena-Francesch and Demirel [140] reviewed the current knowledge on squid ring teeth-based materials, stating they "are an excellent alternative to plastics because they are eco-friendly and environmentally sustainable[biodegradable]." Potential applications of squid 'ring tooth' protein include self-healing fabric, abrasion-resistant coatings, precision drug delivery and smart textiles. The authors are working to make this technology is scalable as they produce these proteins in genetically modified bacteria using a process based on fermentation uses sugar, water, and oxygen to produce biopolymers instead of using the natural squid resources.

3.1.1.2 Agro polymers

Niranjana Prabhu and Prashantha [141] review agro polymers, specifically starch based polymers and thermoplastic starch (TPS) based materials, as biodegradable and eco-friendly packaging material. The authors conclude that essential packaging film properties (barrier, biodegradation, mechanical, and thermal properties) can be improved by using different fillers and changing the source of the starch and call for a multidisciplinary approach to produce better and cheaper eco-friendly materials. Ottesen, Kumar [143] discussed the gas barrier properties of cellulose nanofibrils (CNF) as an environmentally friendly alternative to inorganic and petrochemical coatings of e.g. paperboard in packaging applications. Banerjee, Dick [142] explore carbon dioxide and inedible plant matter as raw material for plastic.

3.1.1.3 Biodegradation

Banerjee, Chatterjee [144] review enzyme mediated biodegradation of various polymers including synthetic, natural and blends of these materials. Zheng, Yanful [145] reviewed technological advancement and microorganisms plastic waste biodegradation. The authors report 1) pro-oxidants and starch are added to synthetic materials to make plastics biodegradable; 2) Thermoplastics derived from polyolefins are able to be biodegraded by photo-degradation and chemical degradation; 3) Thermoset plastics (polyester and polyester

[a] Hawken "maps, measures, models, and describes the 100 most substantive solutions to global warming. For each solution, the authors describe its history, the carbon impact it provides, the relative cost and savings, the path to adoption, and how it works. The goal of the research that informs Drawdown is to determine if we can reverse the buildup of atmospheric carbon within thirty years. All solutions modeled are already in place, well understood, analyzed based on peer-reviewed science, and are expanding around the world."

polyurethane) are attacked by microorganisms due to hydrolytic cleavage of their ester or urethane bonds; 4) microorganisms isolated to utilize polyurethane as a sole source of carbon and nitrogen; and 5) Aliphatic-aromatic copolyesters have commercial applications and are biodegradable. Narancic, Verstichel [146] report that PLA, while not home compostable, becomes home compostable when blended with PCL.

However, Narancic, Verstichel [146] warn that the majority of polymers and blends tested in their study—polymers, polylactic acid (PLA), polyhydroxybutyrate, polyhydroxyoctanoate, poly(butylene succinate), thermoplastic starch, polycaprolactone (PCL), and blends across seven conditions—failed to achieve ISO (Controlled Industrial Composting Conditions) and ASTM (Marine Biodegradation) biodegradation standards, and some showed no biodegradation. And, biodegradation (via thermophilic anaerobic digestion) occurred at 3-6 times commercial plant retention times and with high biogas output.

3.1.1.4 Bioremediation

Animals and bacteria can break down plastics into biodegradable products. Narancic and O'Connor [153] review the advances and possibilities in the biotransformation and biodegradation of oil-based plastics including bio-based and biodegradable polymers, end-of-life management of biodegradables, and a circular economy to reduce plastic waste pollution. New fungi species are biodegrading polyester polyurethane: Pestalotiopsis[147] and Aspergillus tubingensis [148]. Ideonella sakaiensis bacteria breaks PET into terephthalic acid and ethylene glycol in two weeks [149]. Mealworms Larvae can digest Styrofoam in less than 24 h with no cost to survival over 1 month, converting 47.7% of the ingested Styrofoam into CO2 and biodegradable residue[150,151]. Wax moth Galleria mellonella caterpillars can biodegrade polyethylene bags[152].

3.1.2 Making things from waste

Trash, specifically unused containers like bottles, buckets, and tires, can be re-purposed for a variety of profitable items. Individuals (community, entrepreneurs, industry, and researchers) have interest in creating value from trash but need initial support in the form of start-up funds, mentorship, and skill-building, to try out these innovative ideas and enable a circular economy for plastic. Multiple field trials are ongoing in settings where trash contributes importantly to disease. For example, in the indigenous highlands of central Guatemala, a community supported non-profit organization, Long Way Home, is building a vocational school complex from waste materials [154]. Precious Plastics is a global community of hundreds of people working towards a solution to plastic pollution. Knowledge, tools and techniques are shared online, for free[131]. Plastic Bank enables the exchange of plastic for money, items, or Blockchain secured digital rewards, to reveal the value in plastic[23,155]. The Plastic Soup Foundation's campaign of urban mining help companies, educational institutions, governments and communities transition from a linear to a circular economy by managing the whole plastic cycle[156,209]. By partnering with private markets, the profitable recycling of plastic into construction materials will incentivize local entrepreneurs to collect and profit from reused plastic. However, the risk of exposure to contamination [159-161] for entrepreneurs and end users depends on type of materials and the processes used and should be taken into consideration early in the process. We propose these efforts could be strengthened by measuring how these field trials to reduce plastic pollution can also improve health.

3.1.2.1 Waste to Energy

Hawken [130] analyze waste to energy strategies and conclude that for a net implementation cost of $36 billion, we could gain a net operational savings of $19.82 billion and 1.1 gigatons of CO2 reduction. Yet, he warns that this is only a transitional strategy "for a world that wastes too much and needs to reduce its emissions:"

> "Incineration, gasification, and pyrolysis are means of releasing the energy contained in trash. Some of the heavy metals and toxic compounds latent within it are emitted into the air, some are scrubbed out, and some remain in residual ash. With these outcomes, why bother at all? Waste-to-energy plants create energy that might otherwise be sourced from coal- or gas-fired power plants. Their impact on greenhouse gases is positive when compared to landfills that produce methane emissions as organic

> wastes decompose...Even when incineration facilities are state-of-the-art (and many are not), they are not truly clean and toxin-free.”

Several reviews discuss waste to energy regarding, Technological options and challenges, Integrated solid waste management in developing countries, and Environmental impact.

1) **Technological options and challenges:** Kumar and Samadder [162] reviewed the current global scenario of WTE technological options (incineration, pyrolysis, gasification, anaerobic digestion, and landfilling with gas recovery) and challenges. Authors provide a framework for evaluating WTE technological options based on case studies of developed and developing countries (majority unsanitary landfilling) to help the policy makers to understand the current status, challenges and barriers for effective management of municipal solid waste. [163] this study assesses the different waste-to-energy technologies developed to date. This work is divided into four groups: biological treatment of waste; thermal treatment of waste; landfill gas utilization; and biorefineries. Furthermore, integrated solid waste management systems with waste-to-energy technologies are studied and some worldwide examples are provided. Brunner and Rechberger [164] Waste to energy—key element for sustainable waste management. Bosmans, Vanderreydt [165] review thermochemical technologies (incineration, gasification, pyrolysis, plasma technologies, combinations) for energetic valorization of calorific waste streams, with focus on municipal solid waste (MSW), possibly processed into refuse derived fuel (RDF). Authors find: 1) process must be matched to waste; 2) pilot to full scale implementations of plasma gasification/vitrification has shown it extract energy and material value with technical feasibility for MSW/RDF (including excavated waste). Stehlik [166] discuss thermal processing of municipal solid waste (MSW) and achievements: low-NO(x) burners, improved efficiency, heat exchangers, waste heat recovery systems, newly developed equipment for wet scrubbing, dioxin filters and systems for the treatment of sewage sludge. Authors present a new concept for a regional Waste-to-Energy Centre (WTEC) to meet growing population and environmental regulatory standards, using Computational Fluid Dynamics (CFD).

2) **Integrated solid waste management in developing countries:** Marshall and Farahbakhsh [167] Systems approaches to integrated solid waste management in developing countries. urbanization, inequality, and economic growth; cultural and socio-economic aspects; policy, governance, and institutional issues; and international influences have complicated SWM in developing countries. Chattopadhyay, Dutta [168] review Municipal solid waste management in Kolkata, India.

3) **Environmental impact:** Tabasova, Kropac [169] discuss thermal treatment methods for waste-to-energy (WTE) processes technologies performance and impact on environment. Kothari, Tyagi [170] possible future energy utilization patterns and related environmental impacts, potential solutions to current environmental problems and renewable energy technologies and their relation to sustainable development are discussed with great emphasis on waste-to-energy routes (WTERs)

These innovations must come equipped with a knowledge-to-action plan with external stakeholders including existing companies, policy makers and community groups.

3.2 Upstream Policy:

Locally tailored policies can reduce local consumption of single use plastics and require recycling. Researchers should develop and publish relevant policy recommendation and work with MOH at local field sites to develop customized policies to decreased single use plastic supply and trash propagated disease. Already in Kenya, NEMA (National Environment Management Authority www.nema.go.ke) is planning to work with plastic water bottle producers to require recycling.

3.2.1 Governance & policy solutions:

Policy makers are uniquely positioned to prevent and solve public health crises in collaboration with public health officials and communities. Chen, Hwang [102] reported that discarded containers account for 25.4% of

Aedes vector breeding in endemic regions of Taiwan pre-intervention. In 1988, the Waste Disposal Act was amended to make manufacturers, importers, and distributors responsible for the proper recovery, treatment, and recycling of packaging and containers which become an environmental menace. Non-compliance resulted in business suspension. A waste recycling system was established, and a breeding site reduction campaign was promoted for waste management. The authors reported a 98% decreased in dengue incidence reported to the Department of Health from 1988 to 1993. Novak [171] illustrates how Illinois addressed the public health and solid waste problems associated with waste tires, mosquitoes, and mosquito-borne pathogens by developing and implementing Illinois Waste Tire Act. In India, the Prime Minister pledged to eliminate all single-use plastic in the country by 2022 with an immediate ban in urban Delhi[172].

3.2.2 Governance & policy vs community as responsible for public health

Studies place the public health burden on governance policy makers. Ezeah and Roberts [37] reviewed solid waste governance in Nigerian cities from 2008-2010 to compare strategies to enhance waste governance. They conclude that absence of good governance negatively impacted solid waste management performance. individuals resorted to self-help. (1) governance-related barriers constrain sustainable solid waste management (2) public-private partnership is an effective waste management strategy in evolving political systems such as Nigeria (3) mainstreaming the informal sector into the waste management infrastructure in Nigeria will enhance efficiency. Capolongo, Rebecchi [182] define new strategic goals for achieving a "Healthy and Salutogenic City", which will be useful to designers, local governments and public bodies, policy makers, and all professionals working at local health agencies. However, Xanthos and Walker [181] call for an evaluation of effectiveness of bans and levies and recommend more education to reduce demand based on a review of current international market-based strategies and policies to reduce plastic bags and microbeads.

On the edge of community and policy maker responsibility, Tirado-Soto and Zamberlan [35] argue that waste-picker cooperatives[35,178] be strengthened to improve solid waste systems in Rio de Janeiro, Brazil where recycling rates are 45% - 55% yet only 1% of the waste produced is collected selectively by the government. Ferri, Chaves Gde [36] argue that waste pickers should be legally required as stakeholders in the proposed management of municipal solid waste system to solve the challenge of economically managing these wastes.

Norms are also influencing the policy trend. Clapp and Swanston [34] describe the anti-plastic shopping bag norm and policies as a South to North, non-networked and multi-scalar series of events that represent a new environmental norm linked to the influence of material interests in the interpretation of the norm into policy as is described in the case of the US (slow and weak to uptake with strong opposition) and Bangladesh (quick and strong to uptake with weak opposition). Schnurr, Alboiu [177] report public awareness has been triggered by international agencies, organizations, individual and the private-sector to complement or influence legislative interventions. As a result, Single Use Plastic bag interventions (e.g., bans vs. levies) have reduced bag use and SUP pollution 33 - 96%. [177]

Experts call for more policy solutions. In light of increasing frequency and impact of arbovirus outbreaks, Fernandes, Moise [174] highlight issues to address in the global approach to mosquito-borne diseases control. Gyawali, Bradbury [175] describe neglected Australian arboviruses likely to emerge as a result of the Australian Government promoting the development of Northern Australia. They also advocate for surveillance and diagnostic protocols to limit the possibility of large-scale outbreaks. Hotez and Murray [176] urge policy makers responsible for the public health emergency preparedness and the Global Health Security Agenda (GHSA) to consider amending the list of emerging arbovirus diseases.

However, others call for a more community-based approach. Xiao, Zhang [180] conducted a survey in Xiamen, one of eight cities in China which have been operating waste source separation pilot programs since 2000. Institutional factors had the smallest effect on willingness to participate in waste management. Citizens who were better informed and lived in a community/family with more environmentally friendly behaviors have a greater propensity to participate in sustainable waste management. Authors conclude waste policy hierarchy in

Chinese cities and future waste management should change from the current legislative-centered strategy towards locally effective strategies to improve public participation in waste management.

3.3 Downstream Education to decrease demand

3.3.1 Community behavior changes

Creating awareness about the crisis and health and environmental risks surrounding plastic pollution will not decrease supply, but education may increase social pressure and corporate social responsibility to adopt sustainable practices [183,184].
Belontz, Corcoran [186] present a strategy geared towards building successful interdisciplinary collaborations to the "wicked problem" of plastics pollution using a case study of The Synthetic Collective[186], a group of artists, humanities scholars, and scientists. They discuss successes and pitfalls of diverse approaches including social outreach and education.

Education in schools and via public sculpture gardens can create awareness and social pressure to adopt sustainable practices. Hammami, Mohammed [187] Surveyed 400 students from 6 secondary schools in Sharjah city, United Arab Emirates regarding plastic pollution and report 85% of students understand the harm of plastic wastes the environment but with uneven distribution by gender, SES, and age ($P = 0.014$). Most students did not report knowledge of the health impacts of plastics. The authors report all students tended to be involved in the fighting against this dilemma including eco-friendly products, educational campaigns, banning plastic use, voluntary actions, and increase in plastic prices. Eagle, Hamann [185] argue that social marketing principles paired with education and policy can intervene to change behavior to positively impact plastic pollution using a transdisciplinary approach to identify barriers to and enablers of sustained behaviour change.

3.3.2 Community participation in solid waste management

Multiple pilot studies show innovation in involving community in solid waste management. [90,97,99-101,157,179,180,188-193] “I got garbage” is a user-friendly mobile app to promote community based waste management and integration with local governance. [179] Another group in Indonesia promotes community participating in waste management by exchanging recycled plastics for health care insurance. [157]

Sekito, Prayogo [192] compared one community using Community Based Waste Management (CBWM) systems to three communities using typical waste management in Semarang City Indonesia. Using CBWM, households separate municipal solid waste into organic waste and other recyclable materials and the organic waste is composted by the resident’s group. Authors report communities implementing CBWM recycled 33% more solid waste, averting landfill use. Authors report less occurrences of inappropriate waste handling behavior such as backyard burning and throwing waste on the road side or into the river in CBWM communities. Authors report a strong relationship between income and willingness to separate waste and a close correlation between education cooperation.

Following closing of the final dump site, the ban of waste management which causes pollution, illegal dumping and open burning, and the promotion of solid waste reduction at the source, Dhokhikah, Trihadiningrum [189] investigated community participation in household solid waste (HSW) reduction and influencing factors in 300 households in three districts in eastern Surabaya, Indonesia. Authors conclude that four strategies support community participation in HSW reduction: 1) intense HSW reduction training programs; 2) intense information dissemination through mass media and campaign; 3) number of environmental cadres; 4) waste bank optimization. Supporting factors influenced sorting, recycling and composting activities: (a) The level of the respondents’ knowledge consisting of mechanisms of SW sorting, recycle, compost degradable HSW, treat HSW into biogas, and animal feed, definitions of compost and biogas, and the process of recycling and composting; (b) Information from mass media (electronic or print media) about classifying, segregating and handling SW(including decomposable and recyclable wastes); (c) HSW reduction training included activity of educational and trainings of SW sorting, recycling, and composting conducted by local government; private sector or NGOs; (d) Environmental cadre—the present of cadres in the community providing motivation and

counselling in HSW reduction; (e) The availability of waste bank to accept recyclable waste from the customer, and resell the waste to an agent.

In Fiji, the primary vector control emphasis is on source reduction through basic environmental sanitation, health promotion and community participation, backed by law enforcement. Raju [97] evaluated larval source reduction campaign in Fiji using the vector surveillance program of the Ministry of Health. Prior to the intervention, 51% of tires and 21% of drums contained Aedes aegypti. With active community participation (after education and control campaigns, community members protected containers from ovipositioning and collected solid waste for municipal solid waste collection), the number of container habitats for Aedes aegypti was significantly reduced during the nine months.

Tauil and de Azevedo [193] describes community participation in a comprehensive eight-year health program at Porto Nacional, a town in Brazils Amazon region. At the time of the team's arrival, the vermin problem was causing public concern. Work on controlling this problem appeared an appropriate vehicle to achieve the dual objectives of promoting sanitation activities and becoming familiar with and accepted by the community. A health board of volunteers was initiated in Porto Nacional to identify health related problems and propose solutions within the scope of the local government. The board was responsible for improvements in sanitation at the market place and the organization of trash collection. Based on 8 years of program operation, the authors conclude that community participation is an important development resource and that the process of working together is as important as the results achieved.

Sommerfeld and Kroeger [101] summarize methods and insights from a 5-year research and capacity building initiative conducted between 2006 and 2011 in six countries of South Asia (India, Sri Lanka) and South-East Asia (Indonesia, Myanmar, Philippines, Thailand). The initiative, among other things, developed community-based interventions aimed at reducing dengue vector breeding and viral transmission. Where small discarded containers presented the main problem, groups experimented with solid waste management, composting and recycling schemes. Many intervention tools were locally produced, and all tools were implemented through community partnership strategies. All sites developed socially and culturally appropriate health education materials. The study also mobilized and empowered women's, students,' and community groups and at several sites organized new volunteer groups for environmental health.

Tana, Umniyati [100] build an innovative community-centered dengue-ecosystem management intervention in Yogyakarta city, Indonesia and assessed the process and results. The intervention results included: better community knowledge, attitude and practices in dengue prevention; increased household and community participation; improved partnership including a variety of stakeholders with prospects for sustainability; vector control efforts refocused on environmental and health issues; increased community ownership of dengue vector management including broader community development activities such as solid waste management and recycling. The community-centered approach needs a lot of effort at the beginning but has better prospects for sustainability than the vertical "top-down" approach.

Suwannapong, Tipayamongkholgul [90] investigate the associations between environmental factors, dengue knowledge, perception and preventive behaviors of household and collaboration of community members and household risk of dengue in Thailand. High level of community participation in dengue prevention and control in uninfected areas and the information from local authorities and community members reconfirmed that community participation was the key factor against dengue outbreaks. Sustainable process of encouraging community members to eliminate vector breeding sites such as outdoor solid waste disposal is likely to lead to an achievement in dengue prevention and control.

Abeyewickreme, Wickremasinghe [99] assessed if waste management through community mobilization could reduce breeding places at household level and be an effective and sustainable dengue vector control strategy in areas where vector breeding takes place in small discarded water containers. The intervention program included building partnerships of local stakeholders, waste management at household level, the promotion of composting biodegradable household waste, raising awareness on the importance of solid waste management in dengue control and improving garbage collection with the assistance of local government authorities. Waste

management with the elimination of the most productive water container types (bowls, tins, bottles) led to a significant reduction of pupal indices as a proxy for adult vector densities. The coordination of local authorities along with increased household responsibility for targeted vector interventions (in our case solid waste management due to the type of preferred vector breeding places) is vital for effective and sustained dengue control.

Walther, Kunz [188] describe Taiwanese environmental non-governmental organizations (ENGOs) began education campaigns and coastal cleanup events cleanup events and report over a 12-year period that 904,302 items weighing 131,358.3 kg were collected, and 63.6% and 27.2% of items were made of either plastic or plastic mixed with other materials, respectively.

Ruckstuhl [190] report that through monthly voluntary beach cleanups in Famara, 2-3 tons of marine debris have been collected. On an average of about 30 persons, mainly residents from aboard and tourists, participate at each cleanup since 2014 in Famara Beach. The local municipality supports the beach cleanings by providing materials such as bags, gloves, and printing materials. COUP helps organizing and promoting the events, and other local associations take part in the cleanups.

Konecny, Fladmark [191] analyze voluntary cleanups organized by the Great Canadian Shoreline Cleanup (GCSC) along the coast of British Columbia (2013-2016). The majority of recovered litter items were composed of plastic.

Xiao, Zhang [180] conducted a survey in Xiamen, one of eight cities in China which have been operating waste source separation pilot programs since 2000. Over half of respondents were satisfied with local waste management but report waste recycling as inefficient and carried out by the informal sector. Satisfaction was correlated with house prices and higher in newly urbanizing areas compared to old downtown and urban village areas. Willingness to participate in waste management (as estimate by structural equation modeling) was affected by citizen knowledge and social motivation. Institutional factors had the smallest effect. Citizens who were better informed and lived in a community/family with more environmentally friendly behaviors have a greater propensity to participate in sustainable waste management. Authors conclude waste policy hierarchy in Chinese cities and future waste management should change from the current legislative-centered strategy towards locally effective strategies to improve public participation in waste management.
