# Supplementary material for: Solid Wastes Provide Breeding Sites, Burrows, and Food for Biological Disease Vectors, and Urban Zoonotic Reservoirs: A Call to Action for Solutions-Based Research
Source: Front Public Health. 2020 Jan 17;7:405. doi: 10.3389/fpubh.2019.00405 (PMC6979070; doi:10.3389/fpubh.2019.00405)
Supplement: Supplementary file 2 [file Table_1.DOCX]

## Clarifying General Concepts

We classified vector-borne disease as those transmitted biologically by vectors, while urban zoonosis as those associated with wild mammals and domesticated animals of non-agricultural interest such as dogs and cats.

The World Health Organization defines infectious diseases and zoonotic diseases as follows: “Infectious diseases are caused by pathogenic microorganisms, such as bacteria, viruses, parasites or fungi; the diseases can be spread, directly or indirectly, from one person to another. Zoonotic diseases are infectious diseases of animals that can cause disease when transmitted to humans” ^202^.

Urban environments are dynamically defined. For example, in the United States, settlements with 2,500 inhabitants or more are defined as urban, whereas in in Japan, a more densely populated region, only settlements with 30,000 people or more are considered urban ^69^. Urban areas have grown from 34% to 54% of the total population between 1960 and 2014 and are projected to continue growing at 1-2% annually between 2015-2030^72^.

The United Nations Environment Programme published ‘Single-Use Plastics: A Roadmap for Sustainability’ and defines single-use plastics as follows: “often also referred to as disposable plastics, are commonly used for plastic packaging and include items intended to be used only once before they are thrown away or recycled. These include, among other items, grocery bags, food packaging, bottles, straws, containers, cups and cutlery.” ^29^
